# Sex-Equitable Machine Learning Prediction of Ankle Reinjury in Individuals with Chronic Ankle Instability: A 2-Year Prospective Cohort Study with Fairness Audit

**DOI:** 10.1186/s40798-026-01137-3

**Published:** 2026-11-07

**Authors:** Ui-jae Hwang, S. Jun Son, Siu-ngor Fu, Arnold Y. L. Wong, Dustin Bruening, Brent Feland, Matthew Seeley, J. Ty Hopkins

**Affiliations:** 1 Department of Rehabilitation Sciences, The Hong Kong Polytechnic University, Hong Kong, SAR China; 2 Department of Physical Therapy, Yonsei University, Wonju, South Korea; 3 Graduate School of Sports Medicine, CHA University, Seongnam, South Korea; 4 Department of Exercise Sciences, Brigham Young University, Provo, UT USA

**Keywords:** Algorithmic fairness, Ankle sprain, Chronic ankle instability, Machine learning, Prognostic markers

## Abstract

**Background:**

Patients with chronic ankle instability (CAI) face a 2-year reinjury rate approaching 50%, but individualized prediction models that quantify which baseline impairments predict subsequent reinjury within an established CAI cohort remain scarce, and existing prediction models for ankle injury have not assessed sex-related fairness. The aim was to develop and internally validate sex-equitable machine learning (ML) models predicting 2-year ankle reinjury in individuals with CAI, identify the most influential baseline prognostic markers, and audit sex-stratified model performance.

**Methods:**

One hundred physically active individuals (50 male, 50 female) with CAI (mean age 21.8 ± 2.3 years) were recruited from a university population between February 2017 and February 2018 and prospectively followed for 2 years; 96 (49 male, 47 female) with at least one follow-up response were retained for analysis (41 reinjury events, 43% reinjury rate). Fourteen baseline predictors spanning demographic, anthropometric, injury-history, patient-reported, biomechanical, postural-control, and functional-performance domains were collected. Six algorithms were trained with nested cross-validation: an unpenalized logistic regression baseline and five ML algorithms (Elastic Net, support vector machine, random forest, XGBoost, LightGBM). Predictive contribution of each feature was quantified using Shapley additive explanations and partial-dependence plots. A pre-specified fairness audit compared single global and sex-specific Youden thresholds derived out-of-fold using the equalized-odds gap and disproportionate-impact ratio.

**Results:**

All algorithms achieved area under the curve (AUC) values above 0.70; Elastic Net was selected as the primary model (AUC = 0.807, 95% CI 0.714–0.893; calibration slope = 1.015). Sex, arch height index, previous sprains, dorsiflexion range of motion, and Biodex Overall Stability Index consistently emerged as the most influential prognostic markers. Within-sex discrimination was comparable between males and females, but a single global threshold produced an equalized-odds gap of 0.384 that was reduced by 57% (to 0.164) through sex-specific thresholding without retraining the model**.**

**Conclusions:**

ML models with a fairness-audited threshold strategy predicted 2-year ankle reinjury in individuals with CAI, reducing the equalized-odds gap by 57%, though the disproportionate-impact ratio (0.683) remained below the 0.80 benchmark. External validation is required before clinical deployment.

**Supplementary Information:**

The online version contains supplementary material available at https://doi.org/10.1186/s40798-026-01137-3.

## Key Points

- In individuals with chronic ankle instability, sex, arch height index, previous sprains, dorsiflexion range of motion, and Biodex Overall Stability Index consistently emerged as the most influential baseline prognostic markers of 2-year ankle reinjury across multiple machine learning algorithms.
- Under a pre-specified model-selection rule, Elastic Net was selected as the primary model and achieved good discrimination (AUC = 0.807) and near-ideal calibration (calibration slope = 1.015) for 2-year ankle reinjury prediction, comparable to or exceeding existing prospective prognostic tools for chronic ankle instability.
- Applying sex-specific decision thresholds to a single trained model reduced the equalized-odds gap by 57%, providing a practical, low-cost approach to reduce fairness disparities in clinical deployment.

**Supplementary Information:**

The online version contains supplementary material available at https://doi.org/10.1186/s40798-026-01137-3.

## Introduction

Lateral ankle sprain (LAS) is among the most common musculoskeletal injuries in sport, with reported incidence rates of approximately 7 events per 1000 person-years in athletic populations and a higher incidence in females than males (13.6 vs. 6.94 per 1000 exposures) [1, 2]. After a first-time LAS, recovery follows two main trajectories: some patients return to preinjury sport without giving-way or recurrence and are classified as copers, whereas approximately 40% of cases develop chronic ankle instability (CAI) [3, 4]. CAI is characterized by recurrent giving-way episodes, perceived instability, and pathomechanical, sensorimotor, and motor-behavioral impairments that elevate the risk of subsequent injury, including persistent deficits in dorsiflexion range of motion (DFROM), postural control, peroneal strength, and self-reported function [4–6]. These impairments translate into recurrent injury, with reinjury rates approaching 50% in some prospective cohorts and a substantial economic burden through repeated healthcare utilization and lost participation in sport [7, 8].

Several prognostic tools have been developed to predict reinjury or transition into CAI after LAS. The Ankle-GO score, a composite score combining functional tests and self-reported questionnaires, was originally developed and validated as a prognostic tool after LAS [9], and has since been prospectively tested for both 1-year coper classification (area under the curve [AUC] = 0.70) [10], and 2-year reinjury risk, where a low Ankle-GO score was associated with a nine-fold increased risk of recurrence (AUC = 0.75) [11]. In a separate prospective cohort, patient-reported function at return to activity and at 6 months has also been shown to predict recurrent LAS and ankle pain at 12 months [12]. At the candidate-predictor level, previous sprain count, balance deficits, reduced dorsiflexor strength, ankle joint laxity, and altered kinematics have been repeatedly identified as predictors of LAS, with intrinsic risk factors differing between male and female athletes [6, 13, 14]. However, these prognostic tools were developed and validated primarily in early post-LAS cohorts during the return-to-sport phase, not in patients with CAI.

In contrast, CAI studies have been dominated by cross-sectional, case–control comparisons of patients with CAI against healthy controls or copers, which consistently report impairments in postural control, peroneal strength, neuromuscular response timing, dorsiflexion range of motion, foot morphology, and self-reported function [4, 8, 15, 16]. Although such designs efficiently characterize the clinical phenotype, cross-sectional designs cannot establish causal relationships and cannot track which impairments later progress to recurrence [17, 18]. CAI research has therefore been characterized as "model rich, data poor", reflecting that many conceptual models have been proposed but prospective data linking baseline impairments to longitudinal outcomes remain limited [7]. Consequently, the field still lacks prospectively validated, individualized prediction models that quantify which baseline impairments predict subsequent reinjury within an established CAI cohort.

Two complementary advances may address this gap. First, machine learning (ML) offers an analytic framework capable of modeling the non-linear, higher-order interactions among biomechanical, anthropometric, symptomatic, and patient-reported domains. Previous studies have predominantly employed traditional statistical methods that assume linearity and independence among variables, which can limit the modeling of combined risk profiles [19, 20]. When paired with explainable methods such as Shapley Additive Explanations (SHAP) and partial-dependence plots (PDPs), ML can generate interpretable, individualized risk profiles and reveal non-linear thresholds with potential clinical relevance [21]. Second, recent reviews of clinical ML emphasize that high discriminative performance alone is not sufficient. Algorithmic fairness, defined as comparable model performance across demographic groups, has emerged as a complementary requirement for clinically deployed prediction models [22, 23]. Models that perform well on aggregate can still produce differential error rates between male and female patients. Because both LAS incidence and intrinsic risk factors differ between males and females [1, 13], a clinically applicable CAI reinjury prediction model should be audited for sex-related fairness and combined with a mitigation strategy that does not reduce overall predictive performance. To date, no published ML model for CAI reinjury has reported such a fairness audit alongside performance and explainability.

The purposes of this study were (1) to develop and internally validate ML models that predict 2-year ankle reinjury in patients with CAI, integrating routinely obtainable baseline measures across demographic, injury-history, patient-reported, biomechanical, anthropometric, postural-control, and functional-performance domains; (2) to identify the most influential baseline prognostic markers via SHAP analysis and to characterize predictor–reinjury relationships through PDP-derived candidate thresholds; and (3) to audit sex-stratified model performance and quantify the effect of a sex-specific threshold strategy on fairness metrics. We hypothesized that (1) ML models would achieve fair or better discrimination (AUC ≥ 0.70) with adequate calibration; (2) a small set of biomechanical and injury-history markers would emerge as the most influential predictors across multiple algorithms; and (3) within-sex discrimination would be comparable between males and females, but a single global Youden threshold would produce substantial differences in sensitivity and false-positive rates that a sex-specific threshold approach could reduce without retraining the model.

## Methods

### Study Design

This study was a prospective cohort study of individuals with CAI followed for 2 years to identify baseline prognostic markers of ankle reinjury and to develop and internally validate sex-equitable ML prediction models. The study protocol was approved by the Institutional Review Board of Brigham Young University (approval no. F16455), and all participants provided written informed consent before any data collection. The study was performed in accordance with the Declaration of Helsinki. Reporting follows the TRIPOD+AI statement for transparent reporting of multivariable prediction models that use artificial intelligence [24].

### Participants

A convenience sample of physically active individuals with CAI was recruited from Brigham Young University (Provo, Utah, USA) between February 2017 and February 2018. This was a single-center study; all recruitment, baseline assessment, and follow-up surveillance were conducted at this institution. Eligibility criteria followed the recommendations of the International Ankle Consortium for the selection of patients with CAI [25]. Inclusion criteria were (1) a history of at least one significant ankle sprain with the first sprain occurring more than 12 months prior to enrollment and accompanied by inflammatory symptoms and at least one interrupted day of desired physical activity; (2) a history of at least two giving-way episodes in the preceding 6 months; (3) at least five "yes" responses on items 1 to 9 of the modified Ankle Instability Index (AII) [26]; (4) a score of less than 90% on the Foot and Ankle Ability Measure—Activities of Daily Living (FAAM-ADL) subscale; (5) a score of less than 80% on the FAAM-Sport subscale [27, 28]; and (6) a self-reported history of moderate weight-bearing physical activity for at least 3 days per week with a total of at least 90 min per week during the previous 3 months. Exclusion criteria were (1) a history of lower-extremity surgery to musculoskeletal structures; (2) a history of lower-extremity fracture, or (3) an acute musculoskeletal injury to the lower-extremity joints within the previous 3 months. A total of 100 participants (50 male, 50 female) met the eligibility criteria and underwent baseline assessment. Baseline characteristics of the 96 analyzed participants, stratified by 2-year reinjury status, are summarized in Table 1.

**Table 1:** Baseline characteristics by 2-year reinjury status

Variable	No reinjury (*n*=55)	Reinjury (*n*=41)	Test statistic	*p*-value	Effect size (95% CI)
Sex (M/F)	21/34 (38.2% M)	28/13 (68.3% M)	Chi-sq = 8.52	0.004	*h* = 0.61; OR = 3.44 (1.37–8.99)
Age (yr)	21.69 ± 2.30	22.17 ± 2.23	*t* = 1.03	0.307	*d* = 0.21 (−0.19 to 0.62)
BMI (kg/m^2^)	24.26 ± 3.37	24.52 ± 4.25	*t* = 0.33	0.745	*d* = 0.07 (−0.34 to 0.47)
Physical activity (h/wk)	5.81 ± 2.88	5.77 ± 3.21	*t* = −0.07	0.943	*d* = −0.01 (−0.42 to 0.39)
Giving-way episodes (*n*/6 mo)	2.11 ± 1.94	3.39 ± 2.90	*t* = 2.39	0.020	*d* = 0.54 (0.12 to 0.96)
Previous sprains (*n*)	2.91 ± 2.03	4.37 ± 2.51	*t* = 3.05	0.003	*d* = 0.65 (0.23 to 1.06)
AII Q4-8 (*n* yes)	3.24 ± 1.12	3.76 ± 0.99	*t* = 2.40	0.018	*d* = 0.49 (0.08 to 0.90)
FAAM-ADL (%)	85.15 ± 6.43	85.85 ± 4.21	*t* = 0.65	0.517	*d* = 0.13 (−0.28 to 0.53)
FAAM-Sport (%)	69.25 ± 10.70	67.90 ± 9.40	*t* = −0.66	0.513	*d* = −0.13 (−0.54 to 0.27)
Dorsiflexion ROM (deg)	46.48 ± 6.27	42.63 ± 6.52	*t* = −2.91	0.005	*d* = −0.60 (−1.02 to −0.19)
Arch height index	0.36 ± 0.02	0.37 ± 0.02	*t* = 2.82	0.006	*d* = 0.61 (0.19 to 1.02)
SEBT composite (% LL)	96.88 ± 26.59	88.21 ± 6.38	*t* = −2.33	0.023	*d* = −0.42 (−0.83 to −0.01)
BBS-OSI	0.80 ± 0.19	0.91 ± 0.25	*t* = 2.37	0.021	*d* = 0.51 (0.10 to 0.92)
Triple jump (cm/LL)	4.70 ± 1.16	4.90 ± 1.15	*t* = 0.83	0.409	*d* = 0.17 (−0.23 to 0.58)

Values are mean ± SD for continuous variables; counts (column %) for sex. Test statistic: Welch two-sample *t*-test for continuous variables; Pearson Chi-square for sex (df=1). Effect size: Cohen d (95% CI) for continuous variables; Cohen *h* and odds ratio (95% Fisher CI) for sex. *p*-values are two-sided and uncorrected

*AII* Ankle Instability Index (modified) questions 4–8, *FAAM* Foot and Ankle Ability Measure, *ADL* Activities Of Daily Living, *SEBT* Star Excursion Balance Test, *BBS* Biodex Balance System, *OSI* Overall Stability Index, *LL* leg length

### 2-year Follow-Up and Injury Surveillance

**Fig. 1:**
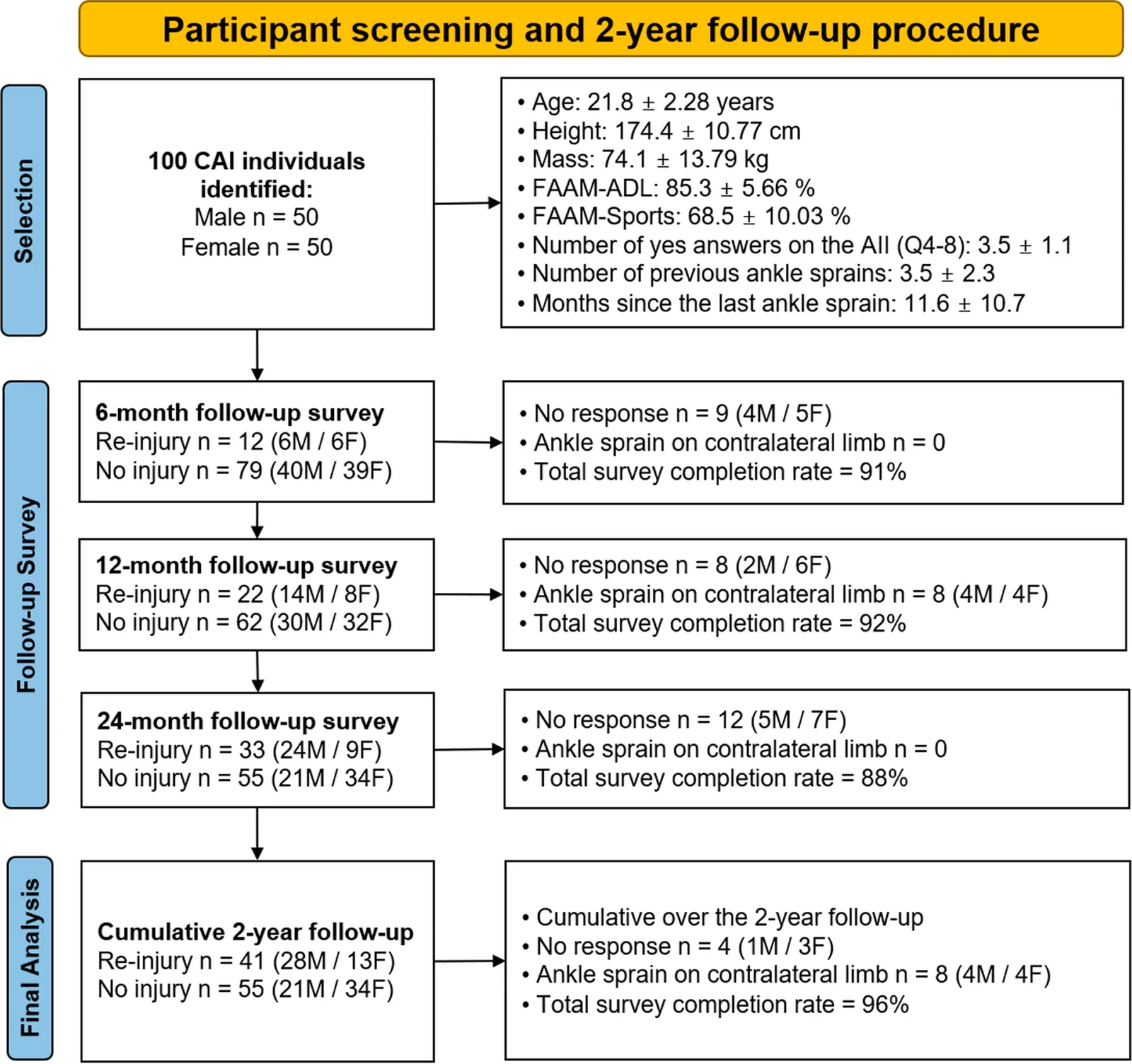
Participant screening and 2-year follow-up procedure. *AII* Ankle Instability Index, *CAI* chronic ankle instability, *FAAM-ADL* Foot and Ankle Ability Measure Activities of Daily Living subscale, *FAAM-Sports* FAAM Sport subscale

The primary outcome was the occurrence of an ankle reinjury on the involved limb during the 2-year follow-up. Ankle reinjury was defined as a new lateral ankle sprain on the same limb assessed at baseline, characterized by a self-reported inversion or supination event [4, 29]. Injury surveillance during the 2-year follow-up was conducted using a Qualtrics online survey (Qualtrics, Provo, UT, USA) administered at three pre-specified time points: 6, 12, and 24 months after the baseline assessment. At each time point, the survey link was distributed via email and mobile text message; participants who did not complete the survey were contacted by telephone and asked to complete it during the call. In addition, participants were instructed to notify the research team by email, text message, or telephone if a new ankle injury occurred between time points, in which case a Qualtrics survey was completed to record the event. Participants who reported a reinjury at any point during the 2-year follow-up were classified into the reinjury group; those who reported no reinjury at all available time points were classified into the no-reinjury group. Contralateral ankle sprains were recorded separately and were not counted as the primary outcome unless the participant also sustained an ipsilateral reinjury. Of the 100 enrolled participants, 4 did not respond at any follow-up time point and were excluded from the final analysis, yielding a final analytic sample of 96 participants (49 male, 47 female) with 41 ankle reinjuries (43% reinjury rate) over 2 years (Table 1 and Fig. 1). Survey completion was 91% (*n* = 91), 92% (*n* = 92), and 88% (*n* = 88) at 6, 12, and 24 months, respectively (Fig. 1). All 96 analyzed participants had their 24-month reinjury status ascertained: all 55 no-reinjury participants completed the 24-month Qualtrics survey (100%), and the 41 reinjury participants had their outcome confirmed by a reinjury event recorded within the 2-year follow-up window. Follow-up completeness was therefore identical in the two outcome groups, and a sensitivity analysis restricted to participants with complete 24-month follow-up would be identical to the primary analysis.

### Baseline Measurements

All baseline measurements were performed by a single trained assessor blinded to the eventual follow-up status. For participants with bilateral CAI, the limb with greater perceived instability was selected as the involved limb. A total of 14 baseline predictors spanning seven domains were collected: demographic and lifestyle (sex, age, habitual physical activity [PA, hours per week]); anthropometric (body mass index [BMI], arch height index [AHI]); injury history and chronic ankle instability symptoms (previous sprains [numbers], recurrent giving-way episodes [Giving-ways], perceived instability [“yes” responses on items 4 to 8 of the modified AII]; patient-reported function (FAAM-ADL, [%] and FAAM-Sport [%]); biomechanical (DFROM [°]); postural control (Star Excursion Balance Test composite reach [SEBT-Composite, % leg length] and Biodex Balance System Overall Stability Index [BBS-OSI]); and functional performance (triple-hop test normalized to leg length [Triple Jump, cm/LL]). All performance-based assessments followed standardized, validated protocols [26, 27, 30–33]. A complete data dictionary of all 14 baseline predictors, including units and detailed acquisition methods, is provided in Supplementary Table 1.

### Procedure

Participants attended two sessions separated by 2 to 3 days: a familiarization session and a testing session. Patient-reported outcomes and demographic information were collected in the familiarization session, followed by randomized clinical assessments in the testing session. Tests performed barefoot included the AHI, SEBT, BBS-OSI, and DFROM measurement. The triple-hop test was performed wearing standardized athletic shoes. For each performance-based test, participants completed three trials with appropriate rest between trials, and the mean of the best two trials was used for analysis. A 3-min rest was provided between tests to minimize fatigue.

### Statistical Analysis

All analyses were performed in R version 4.5.3 (R Foundation for Statistical Computing, Vienna, Austria). Baseline characteristics between the reinjury and no-reinjury groups were compared using t-test for continuous variables and Pearson's Chi-square test for sex. Effect sizes were reported as Cohen's d with 95% confidence intervals (CIs) for continuous variables.

#### Sample Size, Unit of Analysis and Missing Data

The analytic sample of 96 participants with 41 reinjury events corresponded to an EPV ratio of approximately 2.9 for the 14 candidate predictors. Although this is below the conventional EPV ≥ 10 rule, recent simulation evidence indicates that this rule is not universally applicable to penalized regression and tree-based ML models when paired with internal cross-validation (CV) and stability assessments [34–36].

The unit of analysis was the individual participant: each of the 96 participants contributed one modeling record. Performance-based predictors (DFROM, AHI, SEBT-Composite, BBS-OSI, and Triple Jump) were reduced to the mean of the two best of three trials; for bilateral CAI, only the more unstable limb was used; and the outcome was a single binary indicator of any ipsilateral reinjury during the 2-year follow-up. Each participant appeared in one outer test fold per repetition and contributed 5 held-out predicted probabilities that were averaged to a single out-of-fold (OOF) prediction.

Missing values in baseline predictors (< 5% per variable) were imputed by median imputation fit once on each outer training partition and applied to both inner splits and the outer test partition. Feature standardization was refit inside every model-fitting call: separately for each hyperparameter configuration in every inner training split, and once more on the full outer training partition for the final refit (fully nested standardization). Neither procedure used test-fold information, preventing information leakage. Class imbalance was modest (43% reinjury rate) and was addressed through the use of stratified CV rather than oversampling, to preserve calibration.

#### Model Development and Primary Analysis

Six algorithms with different learning approaches were compared. An unpenalized logistic regression served as a pre-specified reference baseline, and five ML algorithms were evaluated: penalized logistic regression (Elastic Net), support vector machine with a radial-basis kernel (SVM), random forest (RF), extreme gradient boosting (XGBoost), and light gradient boosting machine (LightGBM). The baseline logistic regression was fit with base-R glm() under a binomial family with no regularization and was excluded a priori from primary-model selection; its role is to establish whether the ML algorithms provide added value over a standard statistical baseline. The pre-specified primary analysis entered all 14 candidate predictors into each algorithm without external feature selection, relying on each algorithm's internal regularization to handle multicollinearity and noise variables. Predictive performance was estimated using nested CV [37]. The outer loop was stratified 5-fold CV repeated 5 times, performing 25 outer training-test splits; within every outer training partition, the inner loop was stratified 5-fold CV used for hyperparameter tuning by random search (20 configurations per algorithm) optimizing inner-fold AUC. Under this design, each participant appeared in one outer test fold per repetition and contributed 5 held-out predicted probabilities (one per outer repetition), which were averaged to a single participant-level OOF prediction. The full hyperparameter search grid for each algorithm is documented in Supplementary Table 5. The nested CV design is illustrated in Supplementary Fig. 8, and the complete pseudocode, including the exact locations at which each preprocessing step is fitted, is provided in Supplementary Table 6.

#### Performance Metrics

Discrimination was quantified using AUC with 1000 bootstrap-resampled 95% confidence intervals. Calibration was assessed using the calibration slope and intercept and the Brier score. Discrimination and calibration metrics were computed on the averaged OOF predictions of each participant. Operating-point metrics included sensitivity, specificity, precision, negative predictive value, F1 score, MCC, and the Youden index. These threshold-dependent metrics were computed under a fully out-of-sample procedure: within each outer fold, the Youden-optimal threshold was estimated from the training-partition OOF predictions and applied to the untouched test-fold classifications, and each participant's 5 fold-level classifications were then combined by participant-level majority voting. Fold-specific threshold values are summarized as median (IQR) in Table 2. The primary model was selected by a pre-specified rule applied to the five ML algorithms (baseline logistic regression excluded): among the top 3 algorithms by AUC, the model minimizing |calibration slope − 1| +|calibration intercept| was selected, with the lower Brier score as a secondary criterion. The top three algorithms by AUC were retained for explainability and fairness analyses.

**Table 2:** Predictive performance of six algorithms (five machine-learning algorithms and a baseline logistic regression) in the primary analysis without feature selection

Algorithm	AUC (95%CI)	Brier	Cal slope	Cal int	Thr median (IQR)	Sens	Spec	Prec	NPV	F1	MCC	Youden
Logistic regression	0.812 (0.720–0.893)	0.190	0.482	−0.166	0.272 (0.203–0.318)	0.805	0.655	0.635	0.818	0.710	0.456	0.459
ElasticNet*	0.807 (0.714–0.893)	0.182	1.015	0.007	0.368 (0.349–0.406)	0.829	0.618	0.618	0.829	0.708	0.447	0.447
XGBoost	0.805 (0.714–0.888)	0.180	0.766	−0.053	0.358 (0.305–0.437)	0.780	0.618	0.604	0.791	0.681	0.397	0.399
LightGBM	0.799 (0.706–0.886)	0.186	0.600	−0.079	0.415 (0.361–0.581)	0.683	0.764	0.683	0.764	0.683	0.447	0.447
SVM	0.795 (0.700–0.883)	0.187	1.266	0.060	0.438 (0.358–0.494)	0.610	0.764	0.658	0.724	0.633	0.378	0.373
RF	0.747 (0.645–0.841)	0.204	1.895	0.237	0.429 (0.424–0.463)	0.659	0.709	0.628	0.736	0.643	0.366	0.368

AUC and calibration metrics were computed on averaged out-of-fold (OOF) predictions; 95% CIs for AUC are from 1000 bootstrap resamples. Cal slope = 1.0 and Cal int = 0 denote ideal calibration. Threshold-dependent metrics used fold-specific Youden thresholds derived from training-partition OOF predictions and combined by participant-level majority voting across the 5 repetitions; Thr median (IQR) summarizes the 25 fold-specific thresholds. *ElasticNet was selected as the primary model among the top-3 ML algorithms by AUC under a pre-specified rule (minimum |Cal slope − 1| +|Cal int|; ties by lower Brier). Unpenalized logistic regression is listed as a pre-specified baseline and was excluded from primary-model selection

*AUC* area under the receiver-operating-characteristic curve, *Cal* calibration, *CI* confidence interval, *F1* F1 score, *IQR* interquartile range, *MCC* Matthews correlation coefficient, *NPV* negative predictive value, *OOF* out-of-fold, *Prec* precision, *Sens* sensitivity, *Spec* specificity, *Thr* Youden-optimal threshold, *Youden* Youden index

#### Explainability: SHAP and PDPs

Predictive contribution of each feature was quantified using SHAP values, computed on the test predictions of each outer fold and aggregated across folds. Mean absolute SHAP values were used to rank the predictive contribution of each feature under each algorithm, and rank consistency across the top three algorithms was visualized as a heatmap. PDPs were generated for the top features by varying one feature across its observed range while holding all other predictors at their per-participant observed values, then averaging the predicted reinjury probability across participants.

#### Sensitivity Analysis

The sensitivity analysis was designed to test whether the same baseline predictors emerge as informative under a stability-selected subset of the 14 candidates, not to identify a higher-performing model. A drop in discrimination therefore reflects the information lost when predictors are removed. It does not mean that the primary model should have used fewer predictors. Stability selection [38] was performed once per outer training partition using LASSO logistic regression on 100 random subsamples of the outer training set; features with selection probability π ≥ 0.60 were retained, with a top-3 fallback if fewer than three features met this threshold. This design was preferred to nesting the 100-subsample stability procedure inside every inner training split because the 100 internal subsamples already provide an internally cross-validated selection signal, and in our low-EPV setting further partitioning would reduce the per-subsample event count to 13, at which stability selection itself becomes unstable [38]. Per-fold performance with and without feature-selection was compared using the Nadeau and Bengio corrected resampled *t*-test [39, 40], which inflates the variance estimator by a factor of (1/K) + (n_test/n_train) to account for the participant overlap that arises when training partitions share observations across repeated outer folds. The uncorrected paired *t*-test is anticonservative in this setting because the 5 repetitions reuse the same 96 participants. Both the corrected and uncorrected *p*-values are reported for transparency in Supplementary Table 2B, but the corrected *p*-value is treated as the primary inferential statistic.

#### Fairness Audit

A pre-specified fairness audit was performed on the top three algorithms of the primary analysis using sex as the protected attribute. Within-sex discrimination was evaluated by computing AUC separately for male and female participants, with 1000 bootstrap-resampled 95% CIs. Group fairness was quantified under two threshold regimes: (i) a single global Youden threshold, and (ii) sex-specific Youden thresholds derived separately within each sex. To ensure that fairness metrics reflected fully out-of-sample performance, both threshold regimes were derived OOF. For each outer fold, the Youden-optimal threshold was estimated from the training partition's OOF predictions and applied only to the untouched test-fold classifications. Sex-specific thresholds were estimated separately within male and female training-partition predictions. Fairness metrics were then computed on the aggregated test-fold classifications after participant-level majority voting across the 5 outer-CV repetitions. Participant-level bootstrap 95% CIs (1000 resamples) were computed for every fairness metric to expose the instability that arises from the small female reinjury subgroup (13 events). For each regime, the following metrics were computed: [41–43] the EO gap (the maximum of the absolute between-sex differences in sensitivity and false-positive rate), the DP gap (the between-sex difference in positive-prediction rate), and the DI ratio (the ratio of positive-prediction rates between females and males, with values closer to 1.0 indicating greater parity and values ≥ 0.80 considered fair under the four-fifths rule). The full sex-stratified fairness table with bootstrap 95% CIs is provided in Supplementary Table 3. The same fairness audit was repeated on the sensitivity analysis models.

## Results

### Baseline Characteristics

Baseline characteristics are presented in Table 1. Standardized between-group effect sizes identified five continuous baseline variables with moderate effects (|Cohen's d|≥ 0.50, all favoring the reinjury group): previous sprains (*d* = +0.65, 95% CI 0.23–1.06), AHI (*d* = +0.61, 0.19–1.02), DFROM (*d* = −0.60, −1.02 to −0.19), giving-way episodes (*d* = +0.54, 0.12–0.96), and BBS-OSI (*d* = +0.51, 0.10–0.92). Male participants comprised a larger proportion of the reinjury group (68.3% vs 38.2%; odds ratio 3.44, 95% CI 1.37–8.99). AII and SEBT composite reach showed small effects (|d|= 0.42 to 0.49); age, BMI, physical activity, FAAM-ADL, FAAM-Sport, and the triple-hop test showed negligible-to-small effects (|d|≤ 0.21). Full descriptive statistics with 95% CIs are provided in Table 1.

### Predictive Performance of the Primary Analysis

In the pre-specified primary analysis without external feature selection, all five ML algorithms achieved AUC values above 0.70, and the unpenalized logistic regression baseline achieved an AUC of 0.812 (95% CI 0.720, 0.893) (Table 2). Elastic Net achieved the highest ML discrimination (AUC = 0.807, 95% CI 0.714, 0.893), followed by XGBoost (AUC = 0.805, 95% CI 0.714, 0.888), LightGBM (AUC = 0.799, 95% CI 0.706, 0.886), SVM (AUC = 0.795, 95% CI 0.700, 0.883), and RF (AUC = 0.747, 95% CI 0.645, 0.841). Brier scores ranged from 0.180 (XGBoost) to 0.204 (RF). Elastic Net showed near-ideal calibration (slope = 1.015, intercept = 0.007), whereas the baseline logistic regression was substantially miscalibrated (slope = 0.482, intercept = −0.166) and tree-based models exhibited variable calibration (LightGBM slope = 0.600, RF slope = 1.895). Under the pre-specified rule combining discrimination and calibration among the five ML algorithms (baseline logistic regression excluded), Elastic Net was retained as the primary model based on the smallest calibration distance to the ideal (|slope − 1| +|intercept| =|1.015 − 1| +|0.007|= 0.022), and the top three ML algorithms by AUC (Elastic Net, XGBoost, LightGBM) were carried forward for explainability and fairness analyses. Although the baseline logistic regression achieved a slightly higher AUC point estimate than Elastic Net, its poor calibration slope indicates that the added value of the ML models is calibration rather than raw discrimination. The receiver-operating-characteristic curves, calibration plots, and AUC forest plot for all six algorithms are presented in Supplementary Fig. 1.

### Sensitivity Analysis with Feature Selection

The sensitivity analysis incorporating stability-selection feature filtering inside each outer fold showed uniformly lower discrimination across all algorithms (Supplementary Table 2 A): the AUC of SVM, Elastic Net, XGBoost, LightGBM, the baseline logistic regression, and RF decreased to 0.742, 0.735, 0.733, 0.731, 0.727, and 0.717, respectively. Paired per-fold comparisons across algorithms and selected performance metrics are reported in Supplementary Table 2B. Under the Nadeau–Bengio corrected resampled t-test, which accounts for participant overlap across repeated outer folds, none of the per-fold AUC differences between the primary and sensitivity analyses remained statistically significant (all corrected *p* ≥ 0.087), although the uncorrected paired t-test suggested significant differences for four of six algorithms (*p* ≤ 0.033). This reduction in significance illustrates why the corrected test is required when comparing repeated-CV performance estimates. The apparent AUC advantage of the no-feature-selection primary analysis is therefore consistent with, but not statistically confirmed by, the paired-fold comparison. Across the algorithm × fold combinations of the stability-selection procedure, sex (92%), AHI (88%), previous sprains (84%), DFROM (76%), and BBS-OSI (68%) were the only features selected in ≥ 60% of combinations. Receiver-operating-characteristic curves, calibration plots, and the AUC forest plot for the sensitivity analysis are presented in Supplementary Fig. 3. The feature-selection stability heatmap, the consensus-feature bar plot, and the cross-algorithm SHAP rank-consistency heatmap for the sensitivity analysis are presented in Supplementary Fig. 4.

### Baseline Prognostic Markers and Predictor–Reinjury Relationships

**Fig. 2:**
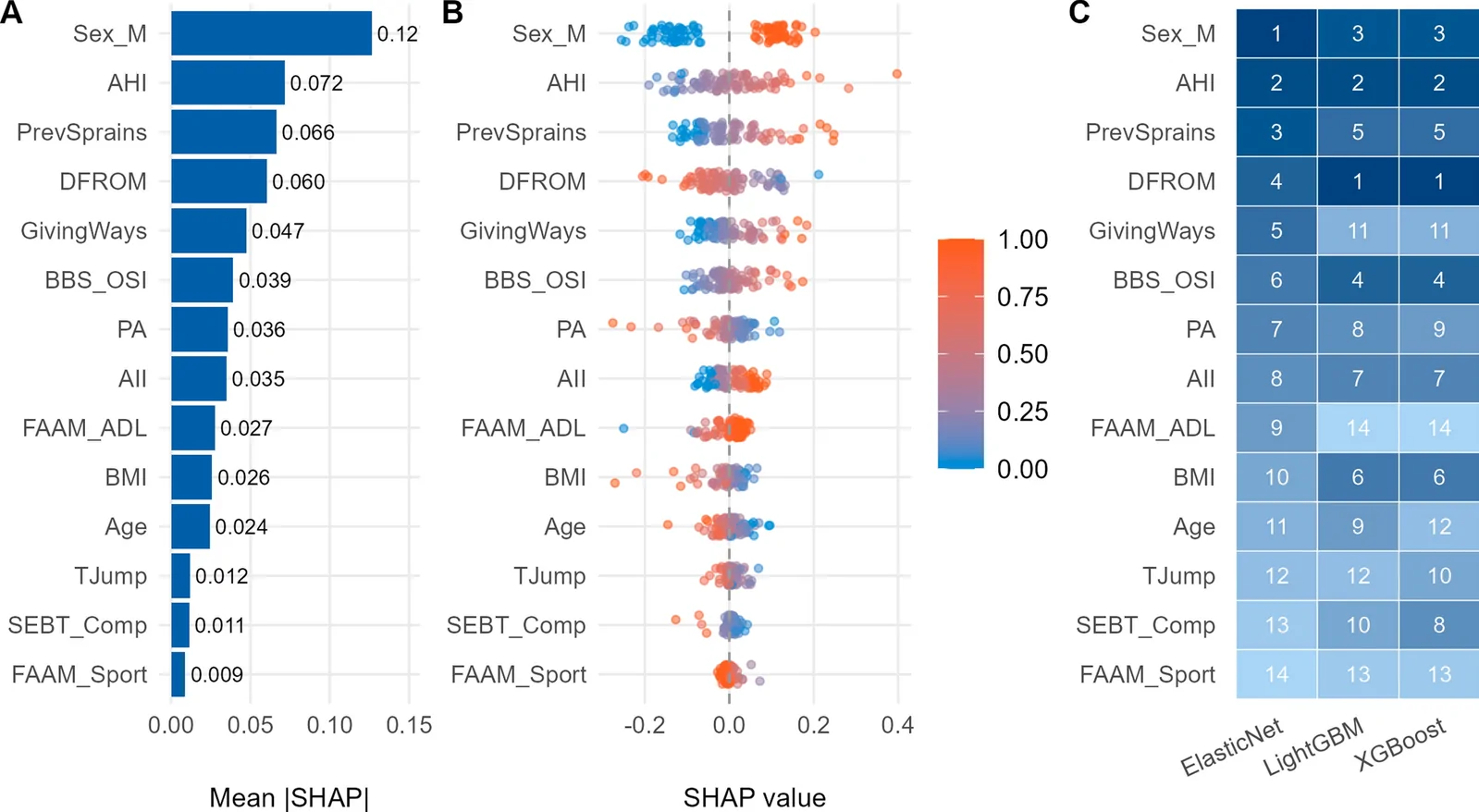
SHAP-based predictive contribution of baseline features in the primary analysis. **A** Mean absolute SHAP values for the ElasticNet primary model, with numeric values shown on each bar. **B** Beeswarm plot of per-participant SHAP values for the ElasticNet primary model; each dot represents one participant, the color gradient indicates the standardized feature value (blue = low, red = high), and horizontal position indicates the direction and magnitude of the SHAP contribution to the predicted reinjury probability. **C** Cross-algorithm rank heatmap of SHAP-based predictive contribution for the three top algorithms of the primary analysis (ElasticNet, XGBoost, LightGBM); cells display the SHAP rank of each feature within each algorithm, with darker shades indicating higher predictive contribution

SHAP-based analysis of predictive contribution in the three top-performing primary models identified a consistent set of five baseline predictors (Fig. 2). Across Elastic Net, XGBoost, and LightGBM, the highest-ranked features were sex, AHI, DFROM, previous sprains, and BBS-OSI, with these five features occupying the top six rank positions across all three algorithms (Fig. 2). Per-participant SHAP distributions for XGBoost and LightGBM are presented in Supplementary Fig. 2. The same top-five predictors emerged from the stability-selection sensitivity analysis, supporting the robustness of these prognostic markers across analytical strategies (Supplementary Fig. 5).

**Fig. 3:**
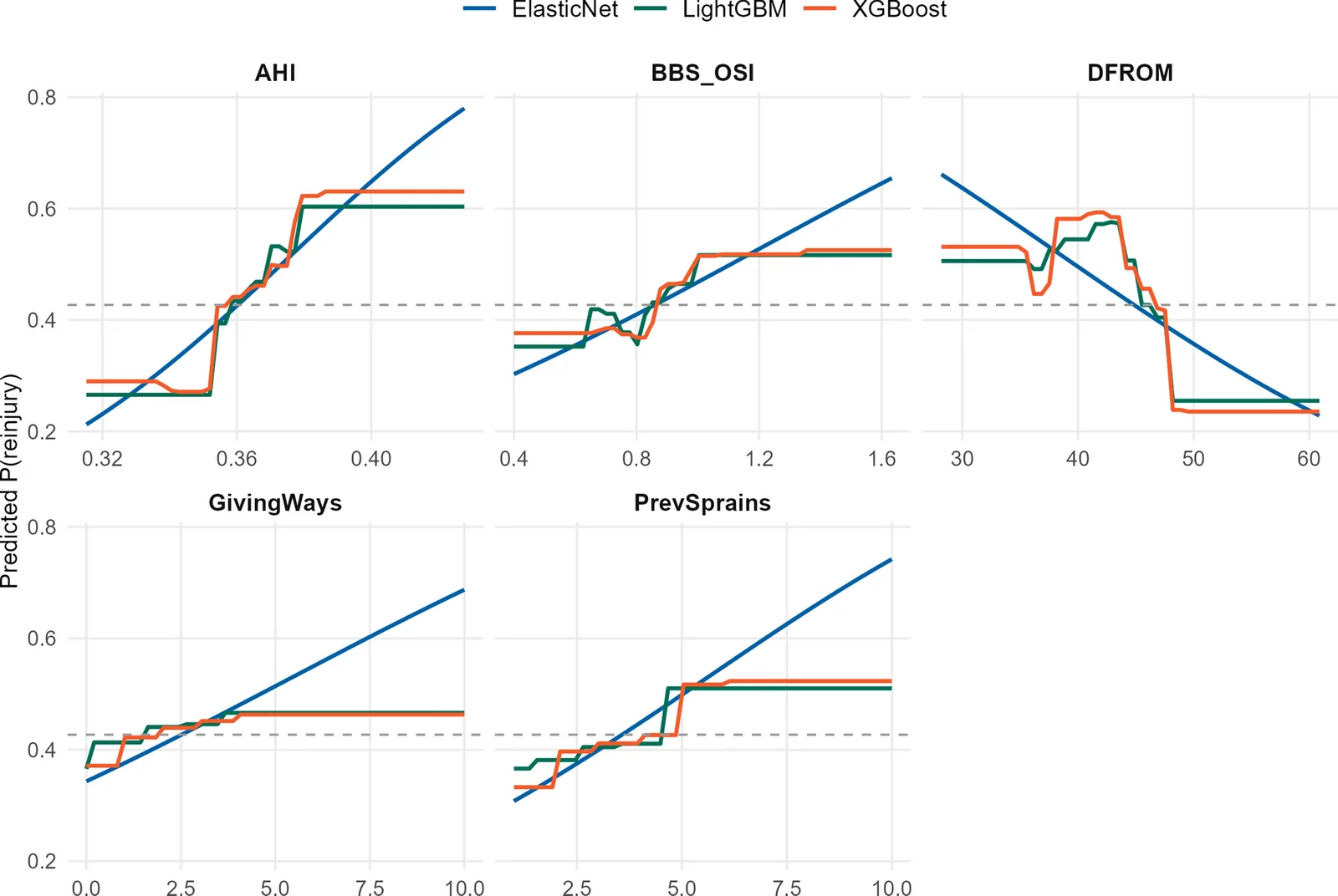
Partial-dependence plots for the top five features in the primary analysis. Predicted reinjury probability as a function of each baseline predictor, holding all other predictors at their per-participant observed values and averaging across participants. Each panel shows the partial-dependence curve for one feature across its observed range, with separate lines for the ElasticNet, XGBoost, and LightGBM primary models. The horizontal dashed line indicates the cohort reinjury rate (0.43). Candidate clinical thresholds were defined as the feature values at which the partial-dependence curve crossed a predicted probability of 0.5

PDPs for the top features (Fig. 3) showed monotonic, predominantly non-linear predictor–reinjury patterns consistent across the three top algorithms. The values at which the marginal partial-dependence curves crossed a predicted probability of 0.5 across the three algorithms were 0.37–0.38 for AHI, 4.7–5.0 for previous sprains, 36–40° for DFROM, and 1.0–1.1 for BBS-OSI. These exploratory model-derived thresholds are presented in Supplementary Table 4. The PDPs for the sensitivity analysis showed similar patterns and are presented in Supplementary Fig. 6.

### Sex-Stratified Fairness Audit

**Fig. 4:**
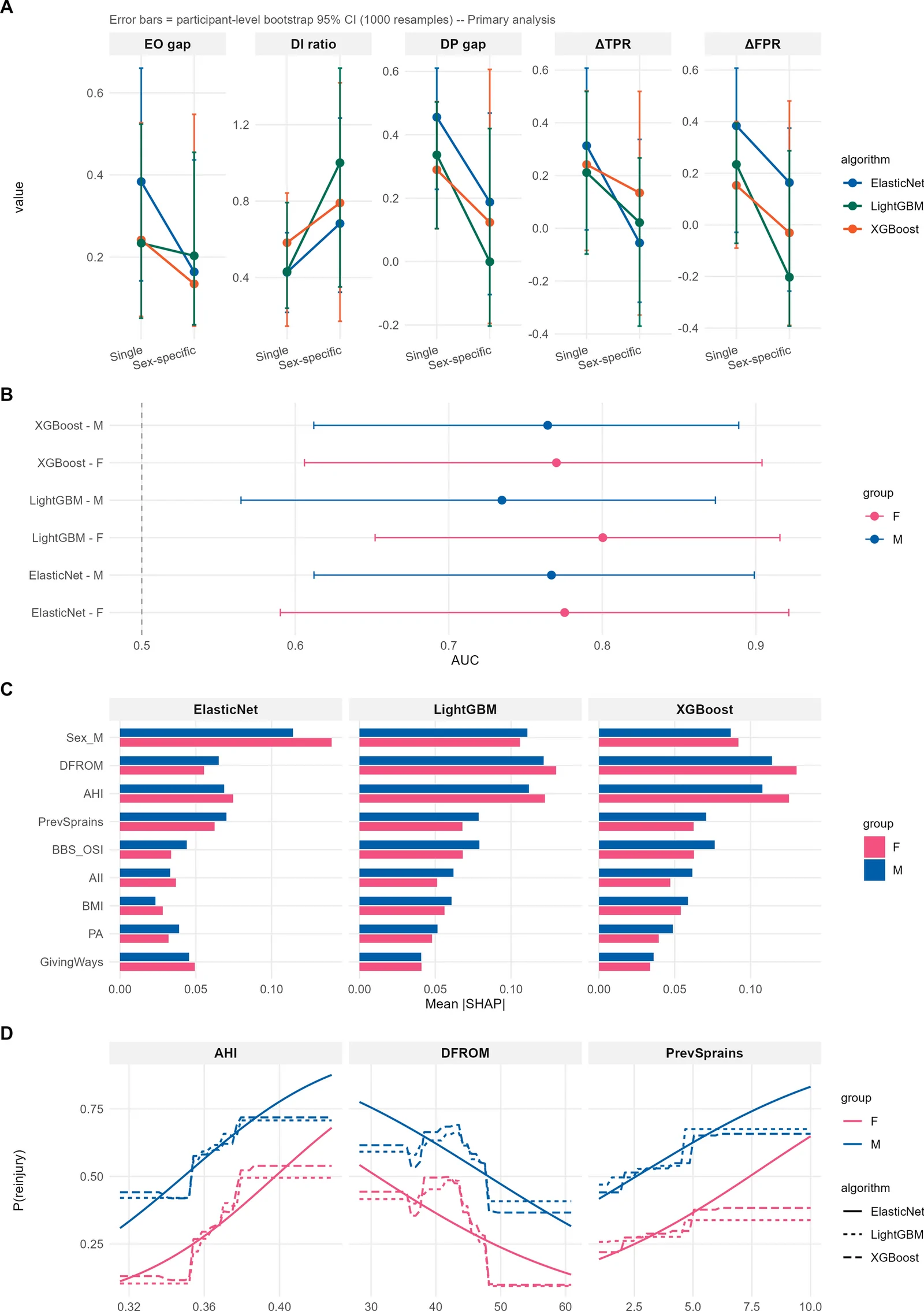
Sex-stratified fairness audit of the primary analysis. **A** Fairness metrics for the three top algorithms (ElasticNet, XGBoost, LightGBM) under single global versus sex-specific Youden thresholds (both derived out-of-fold); error bars = participant-level bootstrap 95% CIs (1000 resamples). **B** Within-sex AUC with bootstrap-resampled 95% CIs for male (M) and female (F) participants. **C** Within-sex mean |SHAP| values for the top predictors. **D** Within-sex partial-dependence plots for the three top predictors (AHI, DFROM, previous sprains). *AHI* arch height index, *AUC* area under the curve, *DFROM* dorsiflexion range of motion, *DI* disproportionate impact; *DP* demographic parity; *EO* equalized odds, *FPR* false-positive rate; *SHAP* SHapley Additive exPlanations

When evaluated within sex, the three top primary models showed comparable discrimination between males and females (within-sex AUC for females: Elastic Net 0.775, XGBoost 0.770, LightGBM 0.800; within-sex AUC for males: Elastic Net 0.767, XGBoost 0.764, LightGBM 0.735), with overlapping 95% CIs across all three algorithms (Fig. 4B). However, when a single global Youden threshold derived OOF was applied, sex-related disparities in operating characteristics emerged. For the primary Elastic Net model at the OOF global threshold, female and male sensitivity were 0.615 and 0.929, respectively, producing an EO gap of 0.384 (bootstrap 95% CI 0.210, 0.670) and a DI ratio of 0.428 (95% CI 0.252, 0.626), well below the 0.80 four-fifths benchmark. Applying sex-specific Youden thresholds derived OOF reduced the EO gap to 0.164 (95% CI 0.044, 0.431), a 57% reduction, and improved the DI ratio to 0.683 (95% CI 0.435, 1.000), without retraining the model. The same pattern was observed for XGBoost (EO gap 0.242 → 0.135, 44% reduction; DI ratio 0.583 → 0.791) and LightGBM (EO gap 0.234 → 0.203, 13% reduction; DI ratio 0.431 → 1.001). Full sex-stratified fairness metrics with bootstrap 95% CIs are reported in Fig. 4A and Supplementary Table 3. The revised DI ratio for the primary Elastic Net model (0.683) remains below the 0.80 four-fifths benchmark, indicating that sex-specific thresholding narrows but does not eliminate the disparity in positive-prediction rates under the present model. The wide bootstrap CIs reflect the small female reinjury subgroup (13 events) and should be considered when interpreting the point estimates. Within-sex predictive contribution patterns from SHAP and PDP were similar between males and females and are presented in Fig. 4C, D, suggesting that the sex-related disparity originated primarily at the decision-threshold stage rather than from sex-divergent predictor effects. The same fairness audit on the sensitivity-analysis models showed a broadly similar pattern, although the improvement in the DI ratio was minimal for SVM (Supplementary Fig. 7).

## Discussion

This prospective cohort study developed and internally validated ML models that predict 2-year ankle reinjury in patients with CAI, and integrated a sex-specific fairness audit into the model evaluation pipeline. The pre-specified unpenalized logistic regression baseline achieved an AUC of 0.812 and a calibration slope of 0.482. The five ML algorithms achieved AUC values ranging from 0.747 to 0.807, with the Elastic Net primary model showing an AUC of 0.807 (95% CI 0.714, 0.893) and a calibration slope of 1.015. A calibration slope of 1.0 indicates ideal calibration. To date, individualized prediction models for ankle reinjury in patients with CAI have been scarce [7], and CAI studies have been dominated by cross-sectional, case–control designs that cannot establish causal relationships between baseline impairments and longitudinal outcomes [4, 17]. The model performance reported here is comparable to or exceeds that of the Ankle-GO score, the only previously published prospective prognostic tool for 2-year recurrent LAS after a single sprain (AUC = 0.75) [11]. The combination of nested CV, bootstrap CIs, and explicit calibration reporting addresses several of the methodological gaps identified in prior CAI prediction literature and supports the validity of the present model [44].

The pre-specified sensitivity analysis applied stability-selection feature filtering inside each outer CV fold to test the robustness of the no-feature-selection primary analysis. Per-fold AUC was consistently lower in the sensitivity analysis across all six algorithms, but under the Nadeau–Bengio corrected resampled t-test, which accounts for participant overlap across repeated CV folds, none of these differences remained statistically significant (all corrected *p* ≥ 0.087), although the uncorrected paired t-test suggested significance for four of six algorithms [40]. The apparent AUC advantage of the primary analysis is therefore best regarded as suggestive rather than confirmatory. Because the primary model uses information from all 14 candidates while the sensitivity analysis uses only the stability-selected subset, the observed drop in discrimination reflects information loss from the smaller predictor set. The sensitivity analysis is therefore a robustness check of the predictor signal, not of the model choice. The same top five predictors emerged from both analyses, with sex (92%), AHI (88%), previous sprains (84%), DFROM (76%), and BBS-OSI (68%) selected in ≥ 60% of the algorithm × fold combinations of the stability-selection procedure. Stability selection was performed once per outer training partition, [38] because further partitioning in this low-EPV setting would have reduced the per-subsample event count to 13 and destabilized the selection itself. The convergence of two analytical strategies on the same predictor set strengthens confidence in the identified prognostic markers.

The five baseline predictors identified by both the primary and sensitivity analyses represent biologically plausible contributors to reinjury risk in patients with CAI. Reduced DFROM has been repeatedly linked to elevated lower-limb injury risk through compromised shock absorption and altered landing kinematics [45–47]. A large prospective cohort of Australian Army recruits identified ankle dorsiflexion range, measured with the same weight-bearing approach as in the present study, as a strong predictor of injury, with restricted dorsiflexion associated with an approximately 2.5-fold higher risk of lower-limb injury [48]. Subsequent work has reported a similar threshold effect for related lower-extremity pathology [46]. The PDP-derived threshold of approximately 35–40° in the present analysis falls within this established range. Elevated AHI, indicative of a supinated foot posture, reduces the contact area between the foot and the ground and shifts the center of pressure laterally, both of which predispose the ankle to lateral inversion sprains [33, 49]. Previous sprain count remains the strongest non-modifiable predictor of subsequent ankle injury [6, 14], and the PDP inflection at approximately five lifetime sprains is consistent with the cumulative-microtrauma model of CAI progression [4]. Higher BBS-OSI values reflect impaired dynamic postural control, a hallmark sensorimotor deficit in patients with CAI [5, 50]. Notably, the triple-hop test was selected in 0% of the stability-selection folds and ranked 12th of 14 in SHAP predictive contribution, suggesting that single hop-based functional tests, although commonly used in return-to-sport batteries, may have limited prognostic value for reinjury in patients with established CAI. These SHAP and PDP results represent predictive contributions to the model prediction rather than causal effects; the five identified markers are best understood as components of a multivariable risk profile. Establishing whether targeted rehabilitation of the modifiable markers (DFROM, dynamic postural control) causally reduces reinjury risk will require follow-up randomized controlled trials.

Sex was identified as the strongest predictive contributor by SHAP analysis (Elastic Net) and was selected in 92% of the stability-selection folds, consistent with prior reports of sex-related differences in CAI risk factors and biomechanics [13, 51]. Within-sex discrimination, however, was comparable between males and females (AUC 0.770–0.800 in females, 0.735–0.767 in males), and the top SHAP predictors and PDP patterns were similar between the two sexes, indicating that the underlying predictor-outcome structure was not sex-divergent. The sex-related disparity in operating characteristics under a single global Youden threshold derived OOF (EO gap = 0.384, DI ratio = 0.428 for Elastic Net) therefore originated primarily at the decision-threshold stage rather than from sex-divergent predictor effects. Applying sex-specific Youden thresholds derived OOF reduced the EO gap by 57% for Elastic Net, with reductions for XGBoost (44%) and LightGBM (13%), without retraining the model. The revised DI ratio for the Elastic Net model (0.683, 95% CI 0.435–1.000) narrowed the disparity but remained below the 0.80 four-fifths benchmark. This pattern is consistent with recommendations in recent clinical ML literature that group fairness should be evaluated and mitigated at the deployment stage when within-group predictive structure is preserved[22, 41]. To date, no published prediction model for CAI reinjury has reported such a sex-specific fairness audit alongside performance and explainability, and the present results provide an initial demonstration that a fairness-audited prediction approach in CAI can substantially reduce sex-related disparity through threshold tuning without sacrificing overall predictive performance, though full parity was not achieved.

From a clinical perspective, the present model offers two distinct utilities. First, the five identified baseline predictors are routinely obtainable in standard outpatient assessment, and the PDP-derived thresholds for AHI (≥ 0.37), DFROM (≤ 37°), previous sprains (≥ 5), and BBS-OSI (≥ 1.0) provide exploratory benchmarks that may help clinicians triage patients with CAI according to anticipated 2-year reinjury risk. Among these predictors, DFROM and dynamic postural control are modifiable through targeted rehabilitation, supporting the prioritization of dorsiflexion mobility work and dynamic balance training over hop-based power training when the goal is reinjury risk reduction [12, 52]. Second, the sex-specific threshold strategy demonstrated here illustrates a practical, low-cost mitigation approach for sex-related disparity: a single set of model coefficients can be deployed across patients of both sexes, with the operating threshold adjusted to the patient's sex at the point of clinical decision-making. This approach contrasts with strategies that require separate models for each sex, which would double the training and validation burden while reducing the effective sample size for each model. These elements support the feasibility of an individualized, fairness-audited risk-stratification approach for use in CAI clinics, although the residual disproportionate-impact ratio (0.683) remains below the 0.80 four-fifths benchmark and warrants further refinement in larger sex-balanced cohorts.

Several limitations should be acknowledged. First, the analytic sample (*n* = 96 with 41 events; EPV ratio 2.9) is limited, and the wide bootstrap CIs for AUC, calibration, and all fairness metrics reflect this uncertainty. The female reinjury subgroup contained only 13 events, so a single misclassification changes within-sex sensitivity by 7.7 percentage points. Although nested CV, bootstrap inference, and a pre-specified sensitivity analysis were used to limit overfitting, external validation in an independent CAI cohort is required before the model can be considered for clinical deployment. Second, the DI ratio under sex-specific thresholds (0.683, 95% CI 0.435–1.000) did not reach the 0.80 four-fifths benchmark, indicating that further methodological work will be needed to achieve full parity in positive-prediction rates. Third, ankle reinjury was ascertained through participant self-report rather than clinician adjudication; misclassification cannot be excluded, although the surveillance approach combining a Qualtrics online survey administered at three pre-specified time points (6, 12, and 24 months), telephone follow-up for non-respondents, and immediate self-report of new ankle injuries was designed to minimize recall bias. Fourth, the PDP-derived thresholds from the model are exploratory and have not been validated against established clinical cut-points; their clinical utility requires further prospective testing. Fifth, the cohort consisted of recreational university-aged athletes, and the present model may not generalize to elite athletes, pediatric populations, older adults, or patients with high-grade structural ankle instability. Sixth, the fairness audit was restricted to sex because no other protected attribute (e.g., race, socioeconomic status, sport type) was systematically recorded; future studies should expand fairness assessment to additional demographic and exposure variables. Finally, candidate predictors did not include strength testing (peroneal, hip), kinesiophobia, or training load, which are additional contributors to reinjury risk and warrant inclusion in future prediction models.

## Conclusion

This prospective cohort study developed and internally validated fairness-audited ML models that predicted 2-year ankle reinjury in individuals with CAI (AUC = 0.807 for the primary Elastic Net model). Sex, AHI, previous sprains, DFROM, and BBS-OSI consistently emerged as the most influential baseline prognostic markers across multiple algorithms and analytical strategies. Within-sex discrimination was comparable between males and females. However, applying a single global Youden threshold produced sex-related disparities in operating characteristics (EO gap = 0.384); applying sex-specific thresholds reduced this gap by 57% (to 0.164) without retraining the model, though the disproportionate-impact ratio (0.683) remained below the 0.80 four-fifths benchmark. These findings identify baseline predictors of subsequent reinjury but do not establish causal relationships; the modifiable predictors identified here (DFROM and dynamic postural control) should be tested in follow-up randomized controlled trials to establish causal evidence for whether their targeted improvement reduces reinjury risk. External validation in an independent CAI cohort is required before the present models can be considered for clinical deployment.

## Supplementary Information

Supplementary Material 1

## Data Availability

The datasets analyzed during the current study are available from the corresponding author on reasonable request.

## Abbreviations

AHI: Arch height index
AII: Modified Ankle Instability Index (items 4–8 used as the perceived instability score)
AUC: Area under the receiver-operating-characteristic curve
BBS-OSI: Biodex Balance System Overall Stability Index
BMI: Body mass index
CAI: Chronic ankle instability
CI: Confidence interval
CV: Cross-validation
DFROM: Dorsiflexion range of motion
DI ratio: Disproportionate-impact ratio
DP gap: Demographic-parity gap
EO gap: Equalized-odds gap
EPV: Events per variable
FAAM: Foot and Ankle Ability Measure
FAAM-ADL: Foot and Ankle Ability Measure—Activities of Daily Living subscale
FAAM-Sport: Foot and Ankle Ability Measure—Sport subscale
LAS: Lateral ankle sprain
LASSO: Least absolute shrinkage and selection operator
LightGBM: Light gradient boosting machine
LL: Leg length
MCC: Matthews correlation coefficient
ML: Machine learning
OOF: Out-of-fold
OR: Odds ratio
PA: Physical activity
PDP: Partial-dependence plot
RF: Random forest
SEBT: Star Excursion Balance Test
SHAP: Shapley additive explanations
SVM: Support vector machine
TRIPOD: Transparent reporting of a multivariable prediction model for individual prognosis or diagnosis
XGBoost: Extreme gradient boosting
