# Supplementary material for: Sex-Equitable Machine Learning Prediction of Ankle Reinjury in Individuals with Chronic Ankle Instability: A 2-Year Prospective Cohort Study with Fairness Audit
Source: Sports Med Open. 2026 Nov 7;12:169. doi: 10.1186/s40798-026-01137-3 (PMC13744576; doi:10.1186/s40798-026-01137-3)
Supplement: Supplementary file 1 [file 40798_2026_1137_MOESM1_ESM.pdf]

Supplementary Material

Sports Medicine – Open

Sex-equitable machine learning prediction of ankle reinjury in individuals with chronic ankle instability: a 2-year prospective cohort study with fairness audit

Running head: *Machine learning prediction of reinjury in CAI*

Authors

Ui-jae Hwang[1,2]; S. Jun Son[3,*]; Siu-ngor Fu[1]; Arnold YL Wong[1]; Dustin Bruening[4]; Brent Feland[4]; Matthew Seeley[4]; J. Ty Hopkins[4]

Affiliations

[1] Department of Rehabilitation Sciences, The Hong Kong Polytechnic University, Hong Kong SAR, China

[2] Department of Physical Therapy, Yonsei University, Wonju, South Korea

[3] Graduate School of Sports Medicine, CHA University, Seongnam, South Korea

[4] Department of Exercise Sciences, Brigham Young University, Provo, Utah, USA

***Corresponding author:** S. Jun Son, PhD, ATC. Graduate School of Sports Medicine, CHA University, Seongnam, South Korea. Email: seongjunson@gmail.com

Supplementary Material

Supplementary Tables

Item	Title
**Supplementary Table 1**	Data dictionary of the 14 candidate baseline predictors
**Supplementary Table 2A**	Predictive performance of the six algorithms in the sensitivity analysis with stability-selection feature filtering
**Supplementary Table 2B**	Paired per-fold comparison of the primary analysis versus the sensitivity analysis across performance metrics, with Nadeau-Bengio corrected p-values
**Supplementary Table 3**	Sex-stratified fairness metrics under single global versus sex-specific Youden thresholds in the primary analysis, with participant-level bootstrap 95% confidence intervals
**Supplementary Table 4**	Candidate clinical thresholds derived from partial-dependence plots in primary analysis
**Supplementary Table 5**	Hyperparameter search grid and reproducibility information for the six algorithms.
**Supplementary Table 6**	Full pseudocode of the four-stage machine-learning pipeline

Supplementary Figures

Item	Title
**Supplementary Figure 1**	Predictive performance of the six algorithms in the primary analysis: receiver-operating-characteristic curves, calibration plots, and AUC forest plot
**Supplementary Figure 2**	SHAP beeswarm plots for XGBoost and LightGBM in the primary analysis
**Supplementary Figure 3**	Predictive performance of the six algorithms in the sensitivity analysis: receiver-operating-characteristic curves, calibration plots, and AUC forest plot
**Supplementary Figure 4**	Feature-selection stability and SHAP rank consistency in the sensitivity analysis
**Supplementary Figure 5**	SHAP beeswarm plots for the top three algorithms in the sensitivity analysis
**Supplementary Figure 6**	Partial-dependence plots for the top features in the sensitivity analysis
**Supplementary Figure 7**	Sex-stratified fairness audit of the sensitivity analysis (out-of-fold thresholds and bootstrap confidence intervals)
**Supplementary Figure 8**	Nested cross-validation design

Supplementary Table 1. Data dictionary of the 14 candidate baseline predictors

Variable	Domain	Type	Unit	Acquisition method
Sex	Demographics	Binary	M / F	Self-report at enrollment
Age	Demographics	Continuous	yr	Self-report at enrollment
BMI	Anthropometric	Continuous	kg/m^2	Standing height and weight measured by trained assessor; BMI = weight / height squared
Physical activity (PA)	Lifestyle	Continuous	h/wk	Self-reported average weekly physical-activity hours over the prior 3 months
Giving-way episodes	CAI symptom	Continuous	n / 6 mo	Self-report count of subjective ankle giving-way episodes in the preceding 6 months
Previous sprains	Injury history	Continuous	n	Self-report lifetime ankle-sprain count
AII Q4-8	CAI symptom	Continuous	0-5	Modified Ankle Instability Index, sum of yes responses to instability items 4-8 of the validated questionnaire
FAAM-ADL	Patient-reported function	Continuous	%	Foot and Ankle Ability Measure Activities-of-Daily-Living subscale; higher = better function
FAAM-Sport	Patient-reported function	Continuous	%	Foot and Ankle Ability Measure Sport subscale; higher = better function
Dorsiflexion ROM (DFROM)	Biomechanical	Continuous	deg	Weight-bearing lunge test with digital inclinometer; mean of best 2 of 3 trials
Arch height index (AHI)	Anthropometric	Continuous	ratio	Arch Height Index Measurement System (archheightindex.com); ratio of dorsum height at 50% of foot length to truncated foot length, mean of best 2 of 3 trials
SEBT-Composite	Postural control	Continuous	% LL	Star Excursion Balance Test composite reach distance (anterior + posteromedial + posterolateral) normalized to leg length, mean of best 2 of 3 trials
BBS-OSI	Postural control	Continuous	score	Biodex Balance System Overall Stability Index, dynamic level 8; mean of best 2 of 3 trials
Triple-hop (cm/LL)	Functional power	Continuous	cm / LL	Triple-hop test, single-leg; total distance of three consecutive hops normalized to leg length, mean of best 2 of 3 trials

All baseline measurements were performed on the involved ankle at the enrollment visit by a single trained assessor blinded to follow-up status. For participants with bilateral CAI, the limb with greater perceived instability was tested. Outcome (ankle reinjury during 2-year follow-up) was ascertained through a Qualtrics online survey administered at 6, 12, and 24 months (telephone follow-up for non-respondents), and immediate self-report at the time of reinjury. CAI, chronic ankle instability; LL, leg length.

Supplementary Table 2A. Predictive performance of six algorithms in the sensitivity analysis with stability-selection feature filtering

Algorithm	AUC (95% CI)	Brier	Cal slope	Cal int	Thr median (IQR)	Sens	Spec	Prec	NPV	F1	MCC	Youden
SVM	0.742 (0.634-0.838)	0.204	1.071	-0.081	0.389 (0.350-0.457)	0.707	0.655	0.604	0.750	0.652	0.358	0.362
ElasticNet	0.735 (0.625-0.829)	0.206	1.003	0.027	0.407 (0.286-0.453)	0.732	0.564	0.556	0.738	0.632	0.294	0.295
XGBoost	0.733 (0.632-0.830)	0.209	0.635	-0.074	0.368 (0.250-0.531)	0.634	0.582	0.531	0.681	0.578	0.214	0.216
LightGBM	0.731 (0.626-0.832)	0.214	0.580	-0.053	0.357 (0.312-0.527)	0.634	0.673	0.591	0.712	0.612	0.305	0.307
Logistic regression	0.727 (0.612-0.825)	0.213	0.644	-0.059	0.388 (0.307-0.477)	0.683	0.691	0.622	0.745	0.651	0.371	0.374
RF	0.717 (0.612-0.814)	0.209	0.991	0.057	0.436 (0.377-0.452)	0.610	0.673	0.581	0.698	0.595	0.281	0.282

Sensitivity analysis: same nested cross-validation pipeline as the primary analysis (Table 2), with stability selection applied inside each outer training partition to derive a reduced predictor subset (features with selection probability $\pi \geq 0.60$ across 100 LASSO subsamples; top-3 fallback if fewer than three features met the threshold). Discrimination (AUC, Brier) and calibration (Cal slope, Cal int) metrics were computed on averaged OOF predictions of each participant. Bootstrap 95% confidence intervals (1000 resamples) are reported for AUC. Threshold-dependent metrics (Sens, Spec, Prec, NPV, F1, MCC, Youden) were computed under the same fully out-of-sample procedure used in the primary analysis: fold-specific Youden thresholds from training-partition OOF predictions applied to untouched test-fold classifications, followed by participant-level majority voting. AUC, area under the receiver-operating-characteristic curve; CI, confidence interval; Cal, calibration; Thr, Youden-optimal threshold; IQR, interquartile range; Sens, sensitivity; Spec, specificity; Prec, precision; NPV, negative predictive value; F1, F1 score; MCC, Matthews correlation coefficient; Youden, Youden index (sensitivity + specificity − 1); OOF, out-of-fold.

Supplementary Table 2B. Paired per-fold comparison of the primary versus sensitivity analysis, with Nadeau-Bengio corrected p-values

Metric	Algorithm	n folds	Primary	Sensitivity	Δ (Sensitivity − Primary)	SD Δ	Corrected p†	Naïve p
AUC	ElasticNet	25	0.804	0.736	-0.068	0.117	0.135	0.007
AUC	XGBoost	25	0.788	0.734	-0.054	0.114	0.203	0.028
AUC	LightGBM	25	0.762	0.732	-0.030	0.108	0.454	0.169
AUC	SVM	25	0.786	0.743	-0.043	0.096	0.269	0.033
AUC	RF	25	0.736	0.717	-0.019	0.088	0.590	0.288
AUC	Logistic	25	0.810	0.727	-0.083	0.130	0.087	0.003
Cal slope	ElasticNet	25	1.089	1.003	-0.086	0.914	0.798	0.641
Cal slope	XGBoost	25	0.451	0.635	+0.184	0.704	0.451	0.204
Cal slope	LightGBM	25	0.467	0.580	+0.113	0.376	0.462	0.148
Cal slope	SVM	25	1.011	1.071	+0.060	0.676	0.782	0.663
MCC	ElasticNet	25	0.629	0.435	-0.194	0.183	0.008	<0.001
MCC	XGBoost	25	0.608	0.419	-0.189	0.287	0.078	0.003
MCC	LightGBM	25	0.581	0.431	-0.150	0.291	0.157	0.017
MCC	SVM	25	0.596	0.431	-0.165	0.169	0.019	<0.001

Nadeau-Bengio corrected resampled t-test with variance-inflation factor 1/K + n_test/n_train applied to fold-level performance differences to account for the non-independence introduced by repeated cross-validation on overlapping training partitions. The ordinary paired t-test on 25 outer folds is anticonservative because the 5 repetitions reuse the same 96 participants, so training partitions overlap substantially and fold-level performance estimates are correlated. The corrected p-value is the primary reported statistic; the naïve p-value is retained for transparency. Negative Δ values indicate that the primary analysis (no feature selection) outperformed the sensitivity analysis (with stability-selection feature filtering) on the same outer folds. Under the Nadeau-Bengio correction, none of the AUC contrasts remained significant at the 0.05 level for the primary versus sensitivity comparison, indicating that the apparent AUC advantage of the primary analysis in the naïve test was inflated by fold-level correlation. Cal slope, calibration slope; MCC, Matthews correlation coefficient.

Supplementary Table 3. Sex-stratified fairness metrics under single global versus sex-specific Youden thresholds in the primary analysis, with participant-level bootstrap 95% confidence intervals

Algorithm	Threshold regime	Sens F (95% CI)	Sens M (95% CI)	EO gap (95% CI)	DP gap (95% CI)	DI ratio (95% CI)	Δ TPR
ElasticNet	Single global (OOF)	0.615 (0.286, 0.923)	0.929 (0.750, 1.000)	0.384 (0.210, 0.670)	0.455 (0.281, 0.629)	0.428 (0.252, 0.626)	+0.314
ElasticNet	Sex-specific (OOF)	0.769 (0.435, 1.000)	0.714 (0.464, 0.929)	0.164 (0.044, 0.431)	0.188 (-0.036, 0.409)	0.683 (0.435, 1.000)	-0.055
XGBoost	Single global (OOF)	0.615 (0.286, 0.923)	0.857 (0.643, 1.000)	0.242 (0.071, 0.549)	0.290 (0.107, 0.475)	0.583 (0.374, 0.830)	+0.242
XGBoost	Sex-specific (OOF)	0.615 (0.286, 0.923)	0.750 (0.464, 0.929)	0.135 (0.029, 0.461)	0.124 (-0.088, 0.352)	0.791 (0.508, 1.137)	+0.135
LightGBM	Single global (OOF)	0.538 (0.222, 0.846)	0.750 (0.500, 0.964)	0.234 (0.102, 0.574)	0.337 (0.150, 0.529)	0.431 (0.230, 0.711)	+0.212
LightGBM	Sex-specific (OOF)	0.692 (0.357, 0.923)	0.714 (0.464, 0.929)	0.203 (0.048, 0.457)	0.000 (-0.223, 0.223)	1.001 (0.667, 1.482)	+0.022

Thresholds derived out-of-fold: for each outer fold, Youden-optimal thresholds were derived from the training partition's out-of-fold predictions and applied only to the untouched test-fold classifications. Sex-specific thresholds were derived separately within male and female training-partition predictions. Fairness metrics were then computed on the aggregated test-fold classifications (participant-level majority vote across the 5 outer-CV repetitions). Bootstrap 95% confidence intervals: participants (row IDs) were resampled with replacement 1000 times; on each bootstrap sample, all fairness metrics were re-computed. Values in parentheses are the 2.5th and 97.5th percentiles across the 1000 replicates. EO gap = max(|Sens_M − Sens_F|, |FPR_M − FPR_F|); DP gap = P(pred=1 | M) − P(pred=1 | F); DI ratio = P(pred=1 | F) / P(pred=1 | M); values closer to 1.0 indicate greater parity, with ≥ 0.80 considered fair under the four-fifths rule. Switching from a single global threshold to sex-specific thresholds reduced the EO gap by approximately 57% (ElasticNet), 44% (XGBoost), and 13% (LightGBM) at the point estimate, but bootstrap CIs are wide because the female subgroup contained only 13 reinjury events. Δ TPR, between-sex difference in true-positive rate.

Supplementary Table 4. Candidate clinical thresholds derived from partial-dependence plots in primary analysis

Feature	Direction	ElasticNet	XGBoost	LightGBM	Consensus
AHI	Higher = more risk	0.373	0.375	0.369	≥ 0.37
DFROM	Lower = more risk	39.695	35.7	35.8	≤ 37 deg
Previous sprains	Higher = more risk	5.028	5.006	4.655	≥ 5
BBS-OSI	Higher = more risk	1.108	0.988	0.996	≥ 1.0
Giving-way episodes	Higher = more risk	4.602	not consistently crossed	not consistently crossed	not consistently crossed

Thresholds correspond to the feature value at which the marginal partial-dependence plot for predicted reinjury probability crosses 0.5. PDPs were generated on the top-3 primary-analysis models (ElasticNet, XGBoost, LightGBM) by varying one feature across its observed range while holding all other 13 predictors at their per-participant observed values and averaging the predicted probability across participants. Where multiple crossings occurred, the lowest-value crossing is reported. Thresholds are model-dependent and should be interpreted as exploratory benchmarks for clinical hypothesis generation, not as validated cut-points. AHI, arch height index; BBS-OSI, Biodex Balance System Overall Stability Index; DFROM, dorsiflexion range of motion.

Supplementary Table 5. Hyperparameter search grid and reproducibility information for the six algorithms

Algorithm	Parameter	Type	Range low	Range high	Sampling
Logistic (baseline)	(no tuning)	—	—	—	—
ElasticNet	alpha	dbl	0	1	uniform continuous
SVM	cost	log	0.1	10	log-uniform
SVM	gamma	log	0.01	1	log-uniform
RF	mtry	int	2	8	uniform integer
RF	min_node	int	3	10	uniform integer
RF	max_depth	int	3	8	uniform integer
XGBoost	nrounds	int	80	300	uniform integer
XGBoost	eta	dbl	0.03	0.2	uniform continuous
XGBoost	max_depth	int	2	5	uniform integer
XGBoost	lambda	dbl	0.5	5	uniform continuous
LightGBM	nrounds	int	80	300	uniform integer
LightGBM	learning_rate	dbl	0.03	0.2	uniform continuous
LightGBM	max_depth	int	2	6	uniform integer
LightGBM	num_leaves	int	7	31	uniform integer

For every algorithm requiring hyperparameter tuning, 20 random hyperparameter configurations were drawn from the ranges specified above (random search) within each outer fold. Configuration selection used mean inner-CV AUC across a 5-fold inner cross-validation of the outer-training partition. Uniform continuous ('dbl') samples uniformly on the natural scale; log-uniform ('log') samples uniformly on the log scale between the two range endpoints; uniform integer ('int') samples uniformly among the integer values in the range. Unpenalized logistic regression (baseline reference) has no tunable hyperparameters and was fit directly on the outer-training partition with base-R stats::glm() and family = binomial().

Preprocessing hierarchy: Missing values in baseline predictors (< 5% per variable) were imputed by median imputation fit once on each outer training partition and applied to both inner splits and the outer test partition. Feature standardization was refit inside every model-fitting call: separately for each hyperparameter configuration in every inner training split, and once more on the full outer training partition for the final refit (fully nested standardization).

Fixed (non-tuned) parameters for each algorithm: Elastic Net (glmnet::cv.glmnet): family = 'binomial', nfolds = 5, type.measure = 'auc'; lambda was selected by the inner-fold CV of cv.glmnet on the standardized outer training partition. SVM (e1071::svm): kernel = 'radial', probability = TRUE, scale = FALSE (standardization was applied externally to preserve the fully nested standardization procedure). Random forest (ranger::ranger): num.trees = 500, probability = TRUE, classification = TRUE, num.threads = 1, internal seed = 42. XGBoost (xgboost::xgb.train): objective = 'binary:logistic', eval_metric = 'logloss', subsample = 0.8, colsample_bytree = 0.8, nthread = 1. LightGBM (lightgbm::lgb.train): objective = 'binary', metric = 'binary_logloss', num_threads = 1. Unpenalized logistic regression (baseline reference) was fit with base-R stats::glm() under family = binomial() and has no tunable hyperparameters.

Software environment: Analyses were performed in R version 4.5.3 (R Foundation for Statistical Computing, Vienna, Austria) on Windows. Package versions: glmnet 4.1-10, ranger 0.18.0, xgboost 3.2.1.1, lightgbm 4.6.0, e1071 1.7-17, pROC 1.19.1.

Random-seed control: A master seed (SEED = 42) governed all stochastic components. Outer folds within repetition r were constructed with seed = SEED × 100 + r; inner folds within outer fold k of repetition r with seed = SEED × 10000 + r × 100 + k; the random hyperparameter grid within outer fold k of repetition r with seed = SEED × 1000 + r × 10 + k; and each bootstrap sample b for AUC 95% CIs with seed = SEED + b. This seeding scheme makes fold construction, hyperparameter search, and bootstrap resampling reproducible across independent runs of the pipeline

Supplementary Table 6. Full pseudocode of the four-stage machine-learning pipeline.

```
Pseudocode

Input: D = participant-level dataset (n=96, 14 predictors, outcome y, sex); R_outer=5;
K_outer=5; K_inner=5; H=20 hyperparameter configs; Alg = {Logistic (baseline),
ElasticNet, SVM, RF, XGBoost, LightGBM}.
STAGE 1: NESTED CV - outer-test probabilities
For each rep r = 1..R_outer:
  Partition D into K_outer stratified folds F[r,1], ..., F[r,K_outer]
  For each outer fold k = 1..K_outer:
    D_train_outer <- D \ F[r,k]  ;  D_test_outer <- F[r,k]
    # REFIT: median imputation on D_train_outer (reused across inner splits)
    For each algorithm alg in Alg:
      Partition D_train_outer into K_inner stratified inner folds
      For each hp config h = 1..H:
        For each inner fold j = 1..K_inner:
          # REFIT: standardization on inner-train partition
          Fit alg(hp=h) on inner-train ;  compute inner AUC on inner-test
        mean_inner_AUC[h] <- mean over inner folds
      hp_best <- argmax_h mean_inner_AUC[h]
      # REFIT: standardization on D_train_outer
      Fit alg(hp=hp_best) on D_train_outer
      Store p[alg, r, k, i] = fitted probability for i in D_test_outer; y[i] = outcome
STAGE 2: Averaged OOF - for discrimination and calibration
For each algorithm alg:
  For each participant i:
    p_avg[alg, i] <- mean over r of p[alg, r, k(i,r), i]       # k(i,r) = fold holding
i in rep r
  Compute AUC[alg], Brier[alg], Cal-slope[alg], Cal-intercept[alg] from (y,
p_avg[alg, ])
STAGE 3: Fully out-of-sample threshold-dependent metrics (Table 2)
For each algorithm alg:
  For each rep r, outer fold k:
    TRAIN_POOL[r,k] <- union over k' != k of { (y[i], p[alg, r, k', i]) : i in F[r,k'] }
# 4 folds
    thr[alg, r, k]  <- argmax_t { sens(t) + spec(t) - 1 } computed on TRAIN_POOL[r,k]
# Youden
    For each participant i in F[r,k]:
      cls[alg, r, k, i] <- 1 if p[alg, r, k, i] >= thr[alg, r, k] else 0
  For each participant i:
# majority vote
    pred_final[alg, i] <- 1 if mean over r of cls[alg, r, k(i,r), i] >= 0.5 else 0
  Compute Sens, Spec, Prec, NPV, F1, MCC, Youden from (y, pred_final[alg, ])
  Report Thr median (IQR) over the 25 fold-specific thresholds { thr[alg, r, k] }
STAGE 4: Fairness audit (top-3 algorithms) - global and sex-specific Youden thresholds
For each algorithm alg in TOP_3:
  # Global regime: as STAGE 3 above
  # Sex-specific regime:
  For each rep r, outer fold k:
    thr_F[alg, r, k] <- Youden threshold on TRAIN_POOL[r,k] restricted to sex = F
    thr_M[alg, r, k] <- Youden threshold on TRAIN_POOL[r,k] restricted to sex = M
    For each participant i in F[r,k]:
      cls_sex[alg, r, k, i] <- 1 if p[alg, r, k, i] >= thr_{sex(i)}[alg, r, k] else 0
  For each participant i:
```

```
Pseudocode
```

```
    pred_sex_final[alg, i] <- majority-vote across 5 repetitions
  Compute Sens_F, Sens_M, EO gap, DP gap, DI ratio with 1000 participant-level bootstrap
95% CIs
```

Pseudocode of the four-stage pipeline described in sections 2.6.1 to 2.6.6 of the Methods. # REFIT comments mark the three refit locations: median imputation on each outer training partition, feature standardization inside every inner training split, and feature standardization on the full outer training partition before the final fit. Majority voting resolves one classification per participant from the 5 fold-level classifications generated across the 5 outer-CV repetitions.

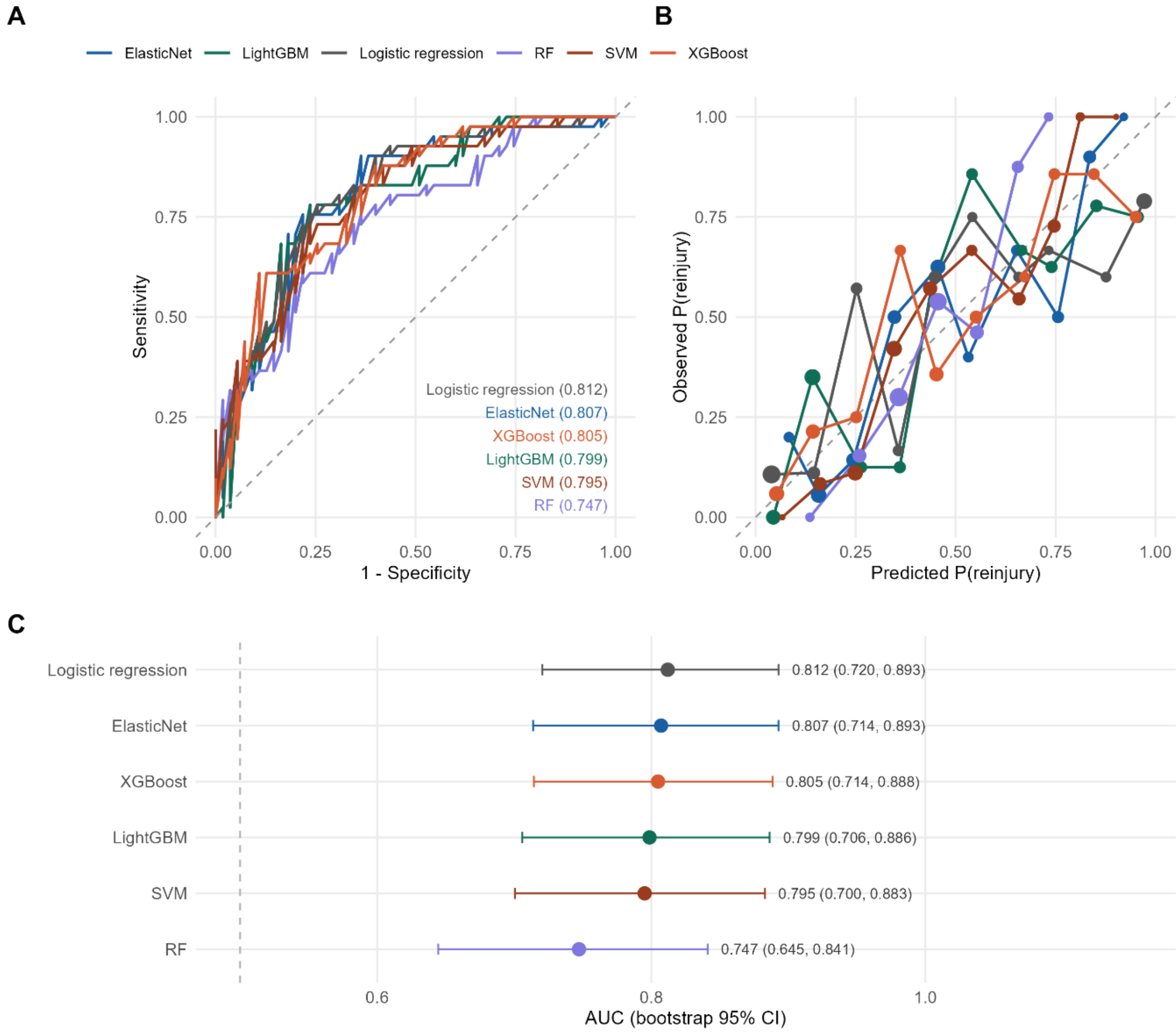

Supplementary Figure 1. Predictive performance of the six algorithms in the primary analysis.

(A) Receiver-operating-characteristic curves on averaged out-of-fold predictions, with AUC in the legend. (B) Calibration plots using decile bins of predicted risk; diagonal = ideal calibration. (C) Forest plot of AUC with bootstrap-resampled 95% CIs (1000 resamples) for the baseline logistic regression and the five ML algorithms.

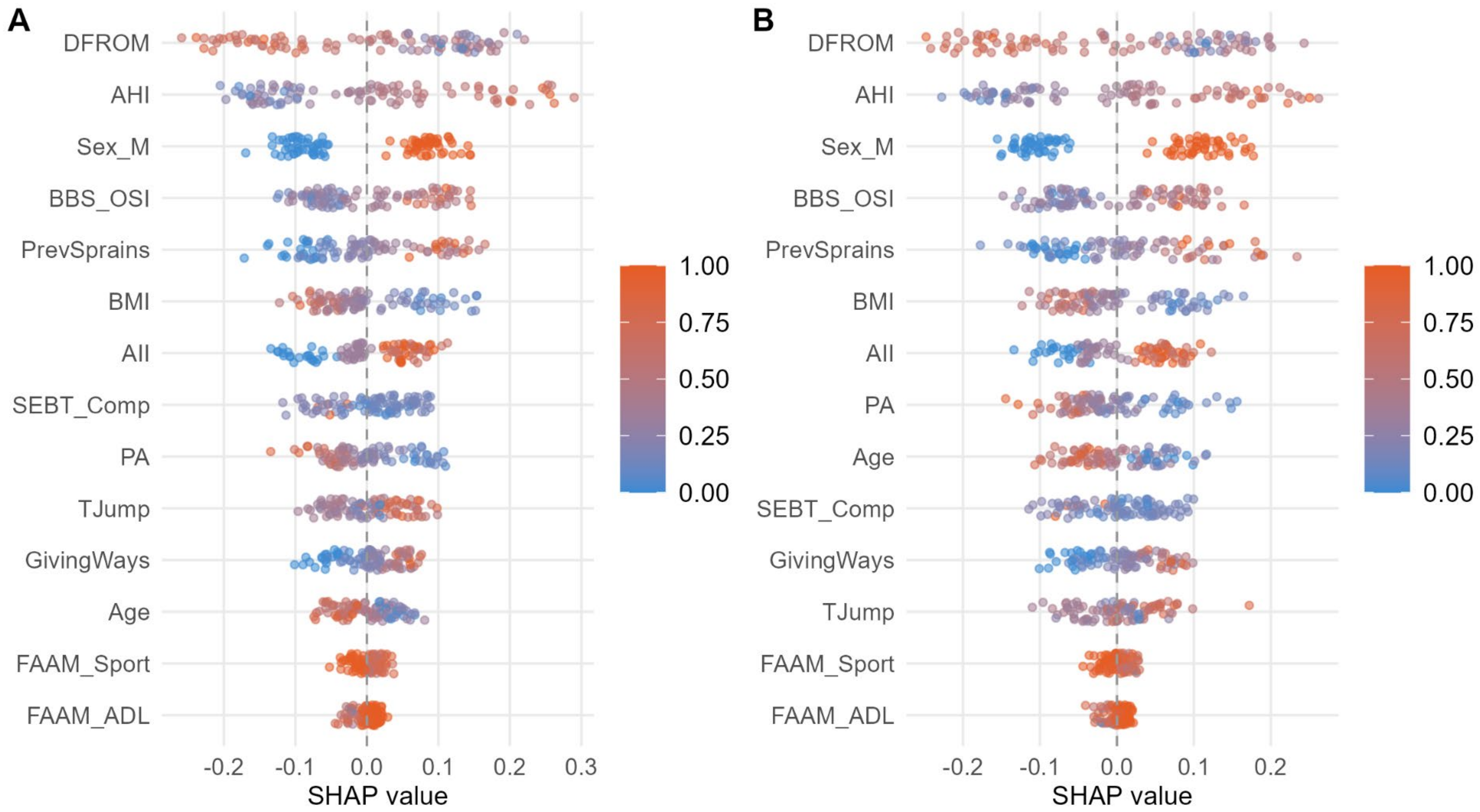

Supplementary Figure 2. SHAP beeswarm plots for XGBoost and LightGBM in the primary analysis.

(A) Per-participant SHAP values for the XGBoost primary model. (B) Per-participant SHAP values for the LightGBM primary model. In each panel, every dot represents one participant, the colour gradient indicates the standardised feature value (blue = low, red = high), and horizontal position indicates the direction and magnitude of the SHAP contribution to the predicted reinjury probability. Features are ordered from top to bottom by descending mean absolute SHAP value.

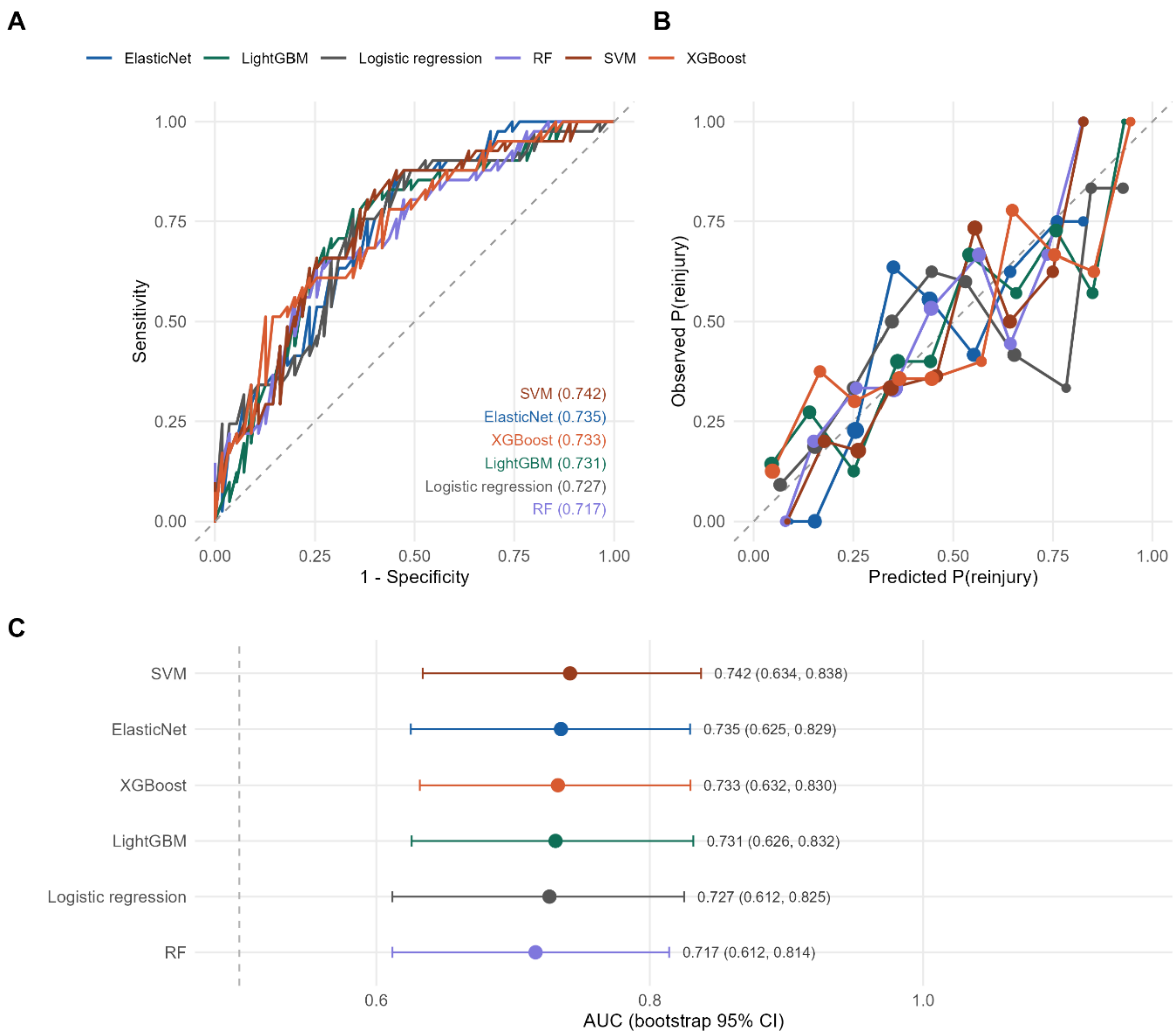

Supplementary Figure 3. Predictive performance of the six algorithms in the sensitivity analysis with stability-selection feature filtering.

(A) Receiver-operating-characteristic curves for the sensitivity analysis (with stability-selection feature filtering), AUC in the legend. (B) Calibration plots; diagonal = ideal calibration. (C) Forest plot of AUC with bootstrap-resampled 95% CIs (1000 resamples).

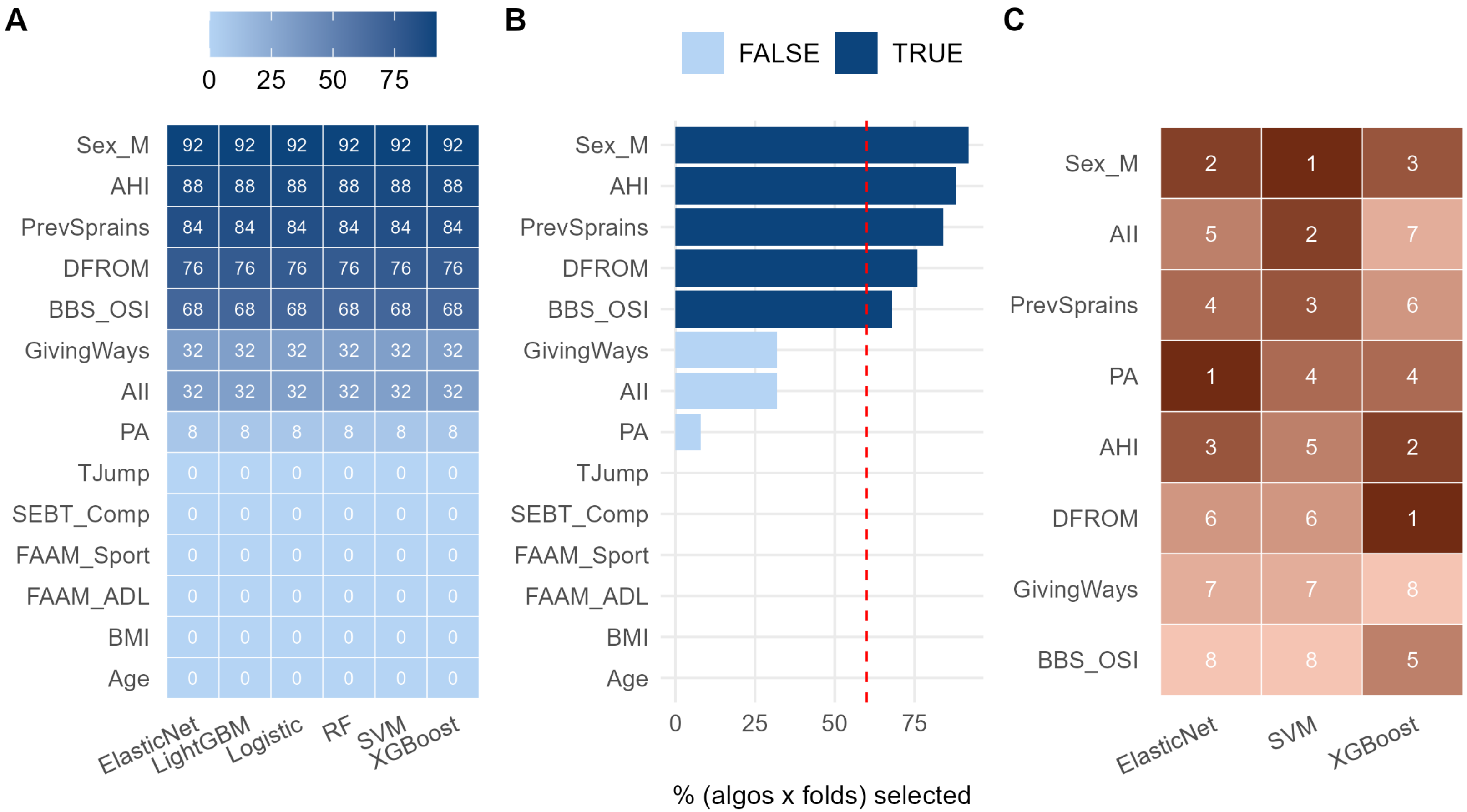

Supplementary Figure 4. Feature-selection stability and SHAP rank consistency in the sensitivity analysis.

(A) Heatmap of the percentage of outer cross-validation folds in which each feature was selected by stability selection (LASSO logistic regression on 100 subsamples; selection probability $\pi \geq 0.60$) within each algorithm. (B) Bar plot of the overall percentage of algorithm × fold combinations in which each feature was selected, with bars coloured to indicate consensus features (selected in ≥ 60% of combinations). (C) Cross-algorithm SHAP rank heatmap for the three top algorithms of the sensitivity analysis (SVM, ElasticNet, XGBoost), showing the SHAP rank of each feature within each algorithm.

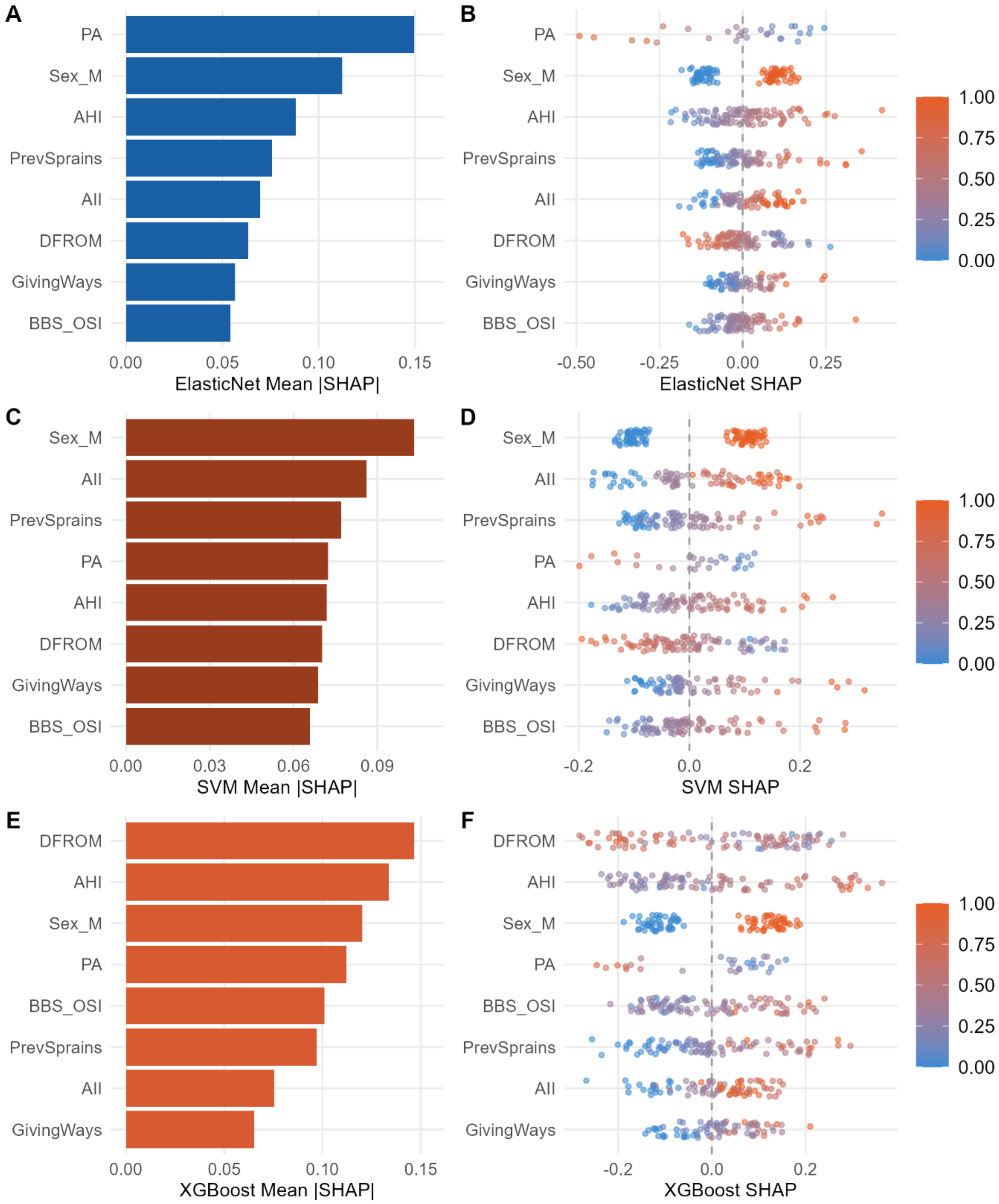

Supplementary Figure 5. SHAP beeswarm plots for the top three algorithms in the sensitivity analysis.

Beeswarm plots of per-participant SHAP values for the three top algorithms of the sensitivity analysis (SVM, ElasticNet, XGBoost), arranged in a two-column layout. Each dot represents one participant, the colour gradient indicates the standardised feature value (blue = low, red = high), and horizontal position indicates the direction and magnitude of the SHAP contribution to the predicted reinjury probability. Features are ordered from top to bottom by descending mean absolute SHAP value within each algorithm.

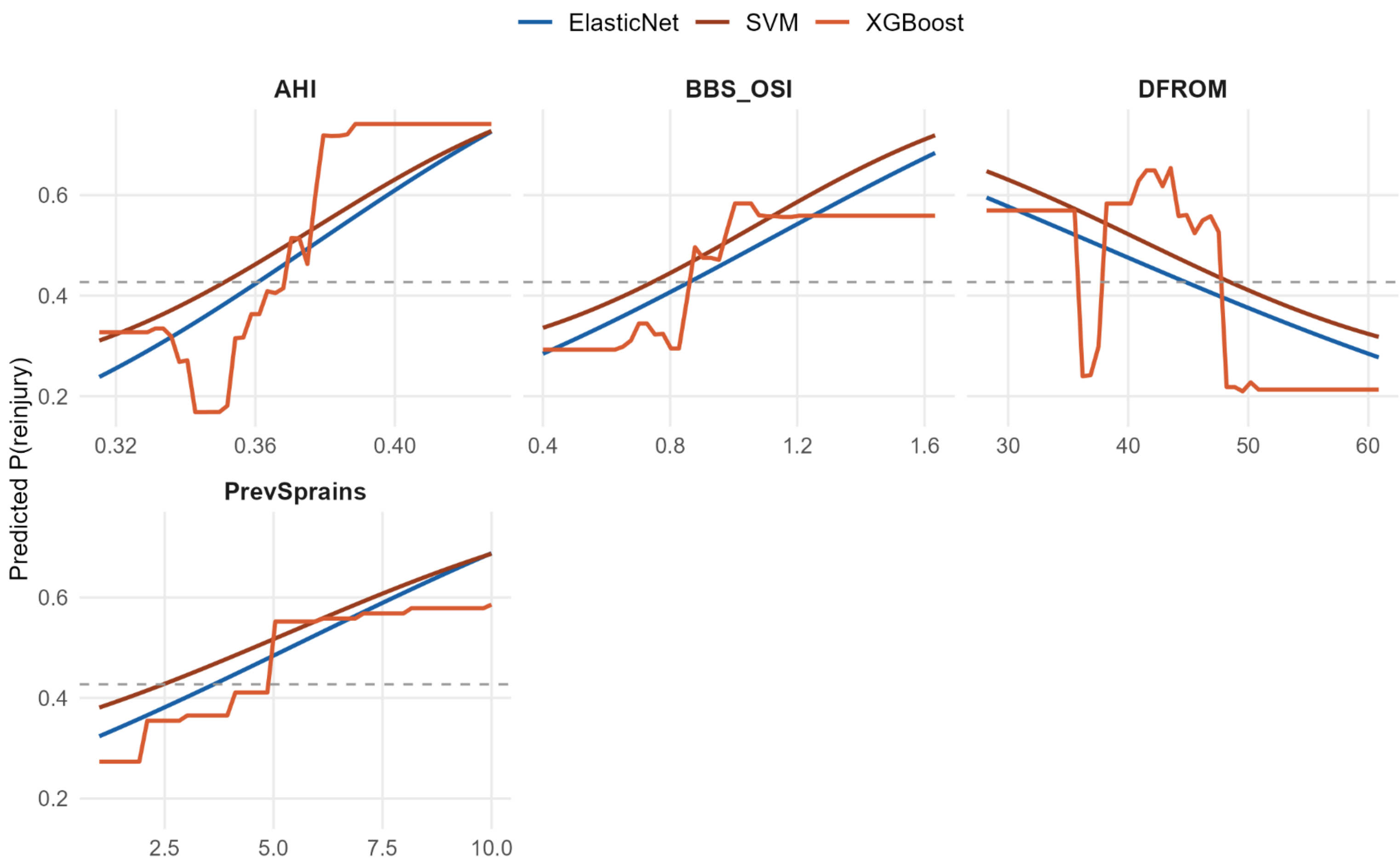

Supplementary Figure 6. Partial-dependence plots for the top features in the sensitivity analysis.

Predicted reinjury probability as a function of each baseline predictor, holding all other predictors at their per-participant observed values and averaging across participants. Each panel shows the partial-dependence curve for one feature across its observed range, with separate lines for the three top algorithms of the sensitivity analysis (SVM, ElasticNet, XGBoost). The horizontal dashed line indicates the cohort reinjury rate (0.43).

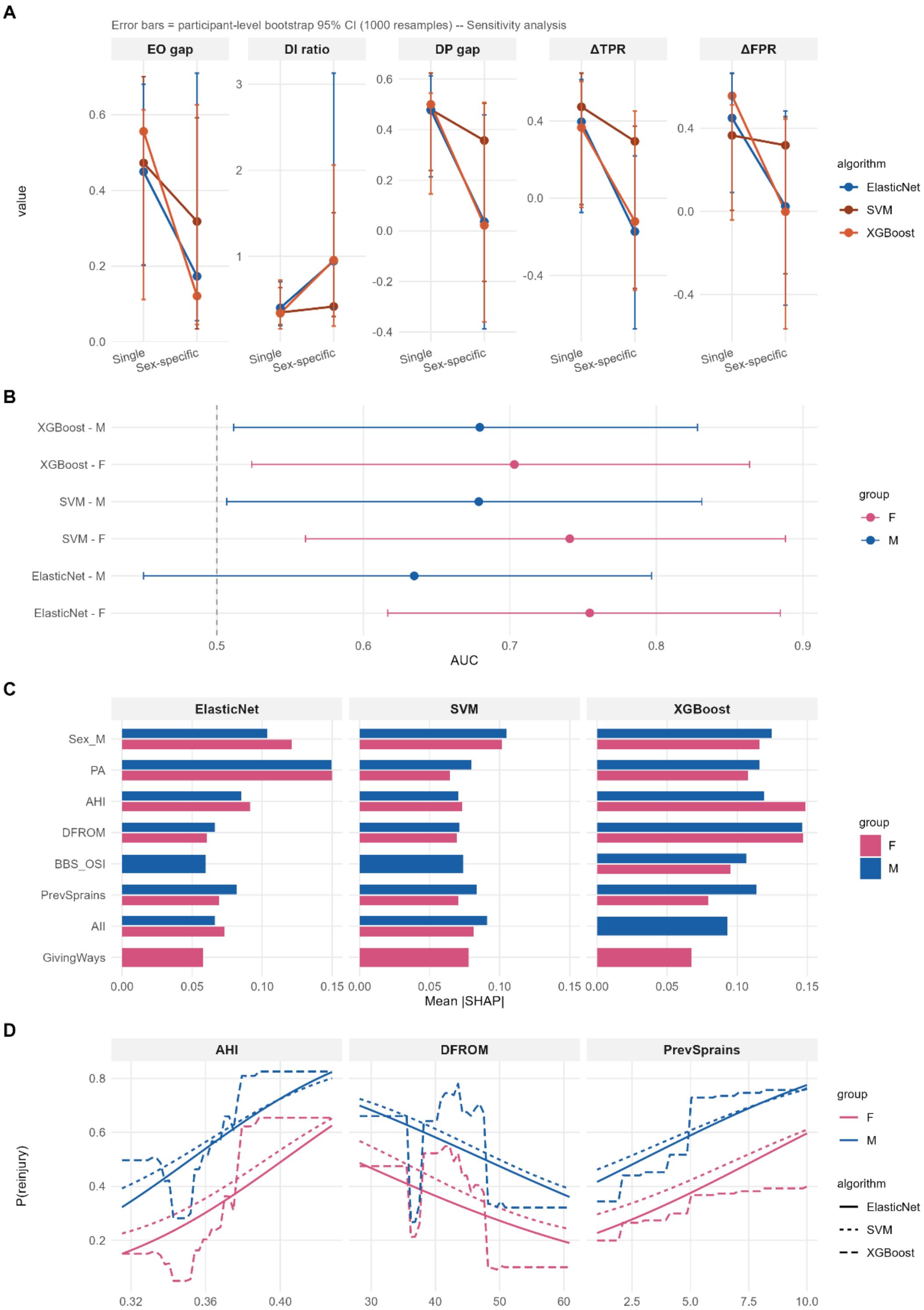

Supplementary Figure 7. Sex-stratified fairness audit of the sensitivity analysis.

(A) Fairness metrics for the three top sensitivity-analysis algorithms under single global versus sex-specific Youden thresholds (both derived out-of-fold); error bars = participant-level bootstrap 95% CIs (1000 resamples). (B) Within-sex AUC with bootstrap-resampled 95% CIs. (C) Within-sex mean |SHAP| for the top predictors. (D) Within-sex partial-dependence plots for the three top predictors. See §2.6.6 for the full procedure.

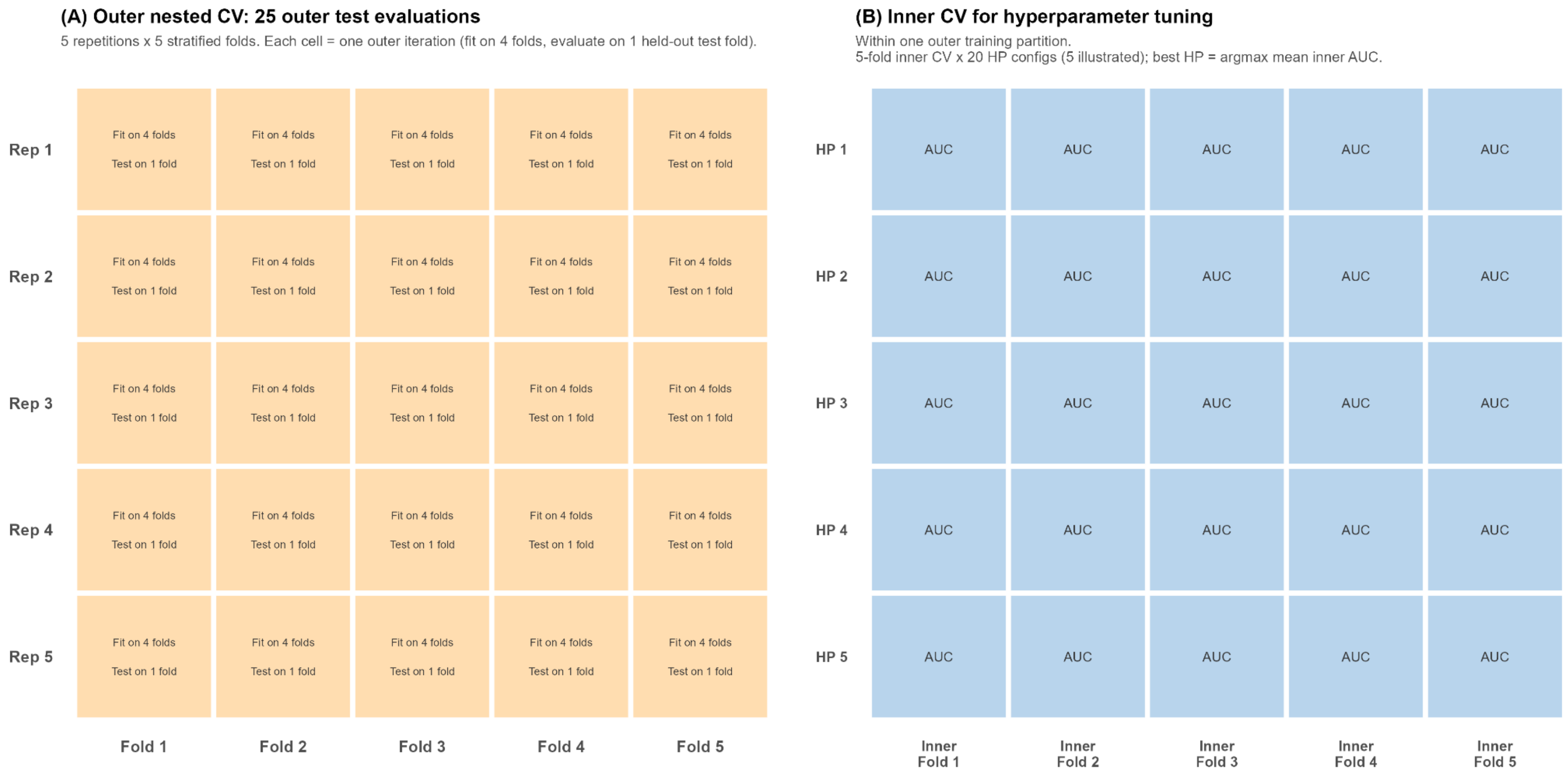

Supplementary Figure 8. Nested cross-validation design.

(A) Outer nested CV: 25 outer test evaluations arranged as a 5 × 5 grid (5 folds × 5 repetitions); each cell = one outer iteration (fit on 4 folds, evaluate on 1 held-out fold). Each participant contributes 5 predicted probabilities that are averaged to one participant-level OOF prediction used for discrimination and calibration. (B) Inner CV within one outer training partition: 5-fold inner CV × 20 hyperparameter configurations per algorithm (5 illustrated); best HP = argmax mean inner-fold AUC.

AUC, area under the receiver-operating-characteristic curve; Brier, Brier score; Cal, calibration; CV, cross-validation; HP, hyperparameter; MCC, Matthews correlation coefficient; NPV, negative predictive value; OOF, out-of-fold; SD, standard deviation.
